# Performance Enhancement of Modified 3D SWCNT/RVC Electrodes Using Microwave-Irradiated Graphene Oxide

**DOI:** 10.1186/s11671-019-3174-9

**Published:** 2019-11-27

**Authors:** Ali Aldalbahi, Mostafizur Rahaman, Mohammed Almoiqli

**Affiliations:** 1 Department of Chemistry, College of Science, King Saud University, Riyadh, 11451 Saudi Arabia; 2 Nuclear Sciences Research Institute, King Abdulaziz City for Science and Technology, Riyadh, 11442 Saudi Arabia

**Keywords:** Composite electrode, Surface chemistry, Morphology, Thermal stability, Electrochemical property

## Abstract

**Abstract:**

The goal of this article is to increase the electrode performance of 3D CNT/RVC electrodes by improving the ease of ion adsorption to and ion desorption from the electrode surfaces. This achievement was done by preparing different composites of synthesized microwave-irradiated graphene oxide (mwGO) with CNT and coated on RVC. The morphology of GO was examined by field emission scanning electron microscopy (FESEM) and X-ray diffraction (XRD) study. Its surface property was checked by energy-dispersive X-ray spectra (EDX), and Fourier transform infrared spectra; whereas, for mwGO by XRD, Raman spectra, and X-ray photoelectron spectra (XPS), which revealed some structural changes of GO after irradiation, where CNTs, being sandwiched between graphene layers, built 3D highly porous architecture inside the electrodes. The electrochemical test of composite electrodes showed increased electrodes conductivity and afforded rapid ions diffusion. It is observed that the 9-CNT/mwGO/RVC composite electrode performed as the best electrode, which showed 29% increment in specific capacitance value compared to the normal CNT/RVC electrode. This best electrode also showed very high cyclic stability in its cyclic voltammetry test that maintained 97% current stability after 2000 cycles, indicating that the electrode can be an effective material for water purification technology.

**Graphical Abstract:**

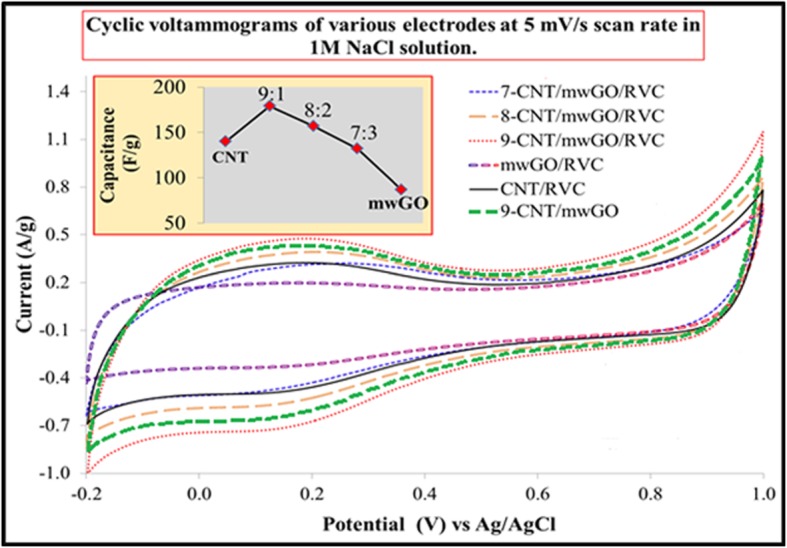

## Introduction

Various carbons like graphene-like carbon flake, multi-walled carbon nanotubes, electrospun fibres, activated carbon cloth, carbon aerogels, reticulated vitreous carbon (RVC), and ordered mesoporous carbon were used as electrodes in the past [2, 4, 47, 51]. These materials have been deemed good electrodes because of their easy accessibility, low cost, resistance to acidic and basic environments, low density, diverse porous structure, and other properties [70]. The use of intrinsically conductive polymer polypyrrole (PPy) and polyethylene dioxylthiophene (PEDOT) as electrode materials is also reported [3, 5, 28].

Graphene material has recently become a material of interest in various applications such as capacitive deionization (CDI), supercapacitor, optical detection, electrochemical biosensing, nanoelectronic circuits, energy, battery, and analytical applications [8, 19, 21, 22, 38, 48, 61, 63]. This is because graphene has an impressive theoretical surface area (2630 m^2^/g) [9, 44], high theoretical specific capacitance (550 F/g) [63, 66], a theoretical electrical conductivity as high as 7200 S/m [36], mean pore diameter and total pore volume reaching to 4.38 nm and 0.44 cm^3^/g, respectively [63], and other interesting properties. These intriguing properties enable graphene to be considered for significant enhancement of carbon nanotube-coated RVC electrodes by acting as conductive bridges for the pores between the carbon nanotube (CNT) particles. Moreover, according to Zhang et al., graphene sheets decreased the aggregation of the CNT bundle and thereby increase the actual accessible area [72]. In addition, the CNT with graphene will build 3D multilayer architecture pores inside the film which are responsible for providing many more available spaces to accommodate ions during electrosorption [30, 71].

CNTs and graphene combinations were used to prepare electrodes for CDI systems [30, 63, 72]. Significant property enhancement was observed in these materials where graphene sheets are intercalated with CNTs to improve the electrons’ transfer amongst the graphene sheets as the salt solutions are inherently resistive. In addition, the spacing between the graphene sheets is increased which improves solution flow and facilitates access of ions to these electrode materials. When these composite electrodes are compared with CNT or graphene electrodes alone, the electrical conductivity, accessible surface area, specific capacitance volumes, electrochemical stability, and electrosorption capacity are improved resulting in low energy consumption for the CDI system. All of them used the Hummer’s method to prepare GO from graphite and were exfoliated by low-power sonication or thermal treatment. These electrodes were made to be used as two-dimensional (2D) sheet electrodes with no significant electrode thickness (0.02 cm).

The CNT/RVC electrode, described in our previous publications [2, 4], was prepared successfully for using as an electrode. This is because it had increased stability at high flow rate pressure, increased the possibility of ions to reach the entire electrode surface in a short time for electrosorption, and shortened the time of ion desorption from the electrode surface. Moreover, we aimed to further increase the electrosorption capacity of CNT/RVC electrode. This can be achieved by firstly improving the pore structure and surface area. To perform that, the electrodes were made as novel three-dimensional (3D) electrodes using RVC electrode as a substrate. In addition, GO nanosheets were synthesized through a modified Hummer’s method and irradiated by microwave to reduce and exfoliate (GO) nanosheets. After that, the microwave-irradiated graphene oxide (mwGO) was dispersed with functionalized CNT in amide solvent (DMF) at various ratios and then used to coat RVC electrodes by a dip-coating method. The sonication time was optimized by monitoring with visible spectra. In addition, the energy of sonication of each milligram of the composite material was selected. The composite electrodes were characterized to check their surface chemistry, morphology, thermal stability, and electrochemical properties. The performance of these electrodes in the system was investigated under different loadings and scan rates.

## Experimental

### Chemicals and Materials

Single-walled carbon nanotubes (SWCNTs) (Hipco-CCNI /Lot # p1001) were procured from Carbon Nanotechnologies, Inc. (Houston, TX), and graphite powder was supplied by Bay Carbon, Inc. *N*, *N*-dimethylformamide (DMF) (AR grade), concentrated nitric acid (HNO_3_ 70%) (AR grade), potassium permanganate (KMNO_4_) (AR grade), ethanol (C_2_H_6_O) (AR grade), and NaCl (AR grade) were procured from Sigma-Aldrich. Moreover, the concentrated sulphuric acid (H_2_SO_4_, 98% w/v) (AR grade), hydrogen peroxide (30% aqueous H_2_O_2_) (AR grade), and concentrated (36% w/v) hydrochloric acid (HCl) (AR grade) were purchased from Univar. The RVC (compressed 60 ppi (pores per inch)) was procured from ERG Materials and Aerospace Engineering. All the above materials were used as received. Membrane filters (0.2 μm pore size GTTP) was supplied by MILLIPORE and cleaned. Milli-Q water (resistivity = 18.2 mΩ cm^−1^) has been used as per requirements.

### Methods

#### Functionalization of CNTs

The functionalization of SWCNT was carried out by refluxing into a round-bottom flask containing 20 mg of it in 40 ml of nitric acid (6 M HNO_3_). The round-bottom was equipped with a magnetic stirrer and immersed in an oil bath at 120 °C for 6 h. The refluxed mixture was then neutralized by washing with water and subsequently with 10 ml of methanol and DMF, separately. Finally, it was dried in an oven at 105 °C for 48 h. The acid-treated SWCNT was designated as a-SWCNT.

#### Synthesis of GO

GO was synthesized by modified Hummer’s method, whose outline was given by Marcano et al. [40]. In this typical method, graphite powder (1 g) was thoroughly mixed with conc H_2_SO_4_ (60 ml) for few minutes. Then, 3.5 g of KMnO_4_ was added in small aliquots so the temperature will not exceed 100 °C. The mixture was continuously stirred for 18 h and then hydrolysed by adding 300 to 500 ml distilled H_2_O (ice bath condition) to form graphite oxide. Thereafter, 30% aqueous H_2_O_2_ (~ 3 ml) was added drop-wise into the mixture till its complete colour changed. Finally, the vacuum-filtrated mixture was washed with HCl solution, distilled H_2_O, and C_2_H_5_OH. The slurried mass was dried under vacuum-oven overnight at 50 °C.

#### Exfoliation and Reduction of GO Using Microwave Irradiation

Exfoliated and reduced GO (mwGO) were formed using a conventional microwave oven (1200 W). After irradiation, the GO glowed red hot accompanied by fuming and sparking, leading to a remarkable volume expansion caused by the violent expulsion of the volatile materials present at the interlayer space of graphene-intercalated compounds [17, 58].

#### Dispersion of mwGO and a-SWCNT

A dispersion of 5 ml containing mwGO 0.1% w/v in DMF, a good solvent for GO dispersions because it can be coupled to an amphiphilic oligoester to produce amphiphilic graphene oxide that is dispersible [13, 17], was prepared using a homogenizer (Branson, Sonifier model S-450D), which was attached with a 13-mm step disruptor horn and a 3-mm tapered microtip. The sonicator was operated by setting the frequency at 20 kHz, amplitude at 25% (means power output 100 W), pulse on-off for 2 and 1 s, and placed on a water-ice bath to prevent overheating of dispersion. Furthermore, functionalized SWCNT 0.2% w/v was dispersed in DMF and optimized. The optimization was done by dispersing a-SWCNT 0.2% w/v in 15 ml DMF using the same sonicator and conditions.

#### Preparation of a-SWCNT/mwGO Composite Coating Solution

The preparation of a-SWCNT/mwGO composite material-coated RVC electrode involves the steps shown in Additional file 1: Figure S1. The dispersion, containing a-SWCNT and mwGO solution, was mixed via probe sonication for 30 min at 30% amplitude (1 s on/2 s off pulse) and bath sonication for 30 min [72] and was optimized for the individual components. The a-SWCNT/mwGO composites were prepared using the weight ratios 90% a-SWCNT to 10% mwGO, 80% a-SWCNT to 20% mwGO, and 70% a-SWCNT to 30% mwGO which were the weight ratios optimized in our laboratory, one of which (90% a-SWCNT to 10% mwGO) afforded the highest specific capacitance for the composite material in an electrode and that also gave the highest capacity for electrosorption. This has been discussed later on in the “Results and Discussion” section.

#### Pre-treatment of the RVC Electrode

The best pores per inch (ppi) RVC electrode, used as a substrate for loading carbon material, was optimized (see Additional file 1: Figure S2). It optimized one was 60 ppi RVC electrode because of its increased conductivity that readily allows the movement of electrical charge and had comparatively higher capacitance and surface area. Therefore, this optimized electrode was chosen as a substrate for loading a-SWCNT/mwGO composite material to be used as an electrode in a CDI system. Definite sizes of electrodes (length 4 cm × width 1.8 cm × thickness 0.3 cm) (2.16 cm^3^) were taken by cutting from a block of RVC and were soaked in 2 M HNO_3_ for 24 h to remove any impurity. The electrodes were washed several times with distilled water to remove the acid. The effluent’s pH was checked regularly until it became neutral. The removal of organic impurity from the electrode was carried out by soaking in CH_3_OH for 2 h. The electrode was then dried under N_2_ flow and followed by heating in an oven overnight at 110 °C. The dried electrodes were weighed properly through a suitable balance.

#### a-SWCNT, mwGO, and a-SWCNT/mwGO Composite Dip-Coated RVC Electrodes

All electrodes which are a-SWCNT, mwGO, or a-SWCNT/mwGO composites with RVC were made by the dip-coating method by slowly immersing into the composite solution. Additional file 1: Figure S3 schematically shows the dip-coating process of RVC in a composite solution of 9 CNT: 1 mwGO. The dip-coated substrates were initially placed in an oven for 2 h for drying at 100 °C and later on in a vacuum oven for 2 h at 50 °C for removing all organic solvents that remained in the micropore of the electrodes.

Table 1 presents the weights of material loaded on the RVC electrodes. It was calculated by taking the weight of electrode before and after its dip-coating. The 9 a-SWCNT/mwGO composite material weights coated on RVC electrodes were 10, 30 and 50 mg, and all the other composite material weights coated on RVC electrodes were 50 mg including a-SWCNT- and mwGO material-coated RVC electrodes. A maximum of 50 mg loading was used because our previous work [2, 4] showed that 50 mg was the highest amount of CNT material that can be loaded into the RVC electrode, and it afforded the highest capacitance and electrosorption capacity in terms of geometric volume. Initial investigations were done (Table 1 (a)) to determine which electrode material gave the best performance with maximum of 50 mg loading. Subsequent experiments (Table 1 (b)) were performed on the best electrode to confirm that 50 mg loading indeed gave the best result.

**Table 1:** Details of carbon materials coated on RVC (2.16 cm^3^) electrodes

samples	Ratio in coating solution		Material weight (mg)
	a-SWCNT	mwGO	
(a)			
CNT/RVC	10	0	50
9-CNT/mwGO/RVC	9	1	50
8-CNT/mwGO/RVC	8	2	50
7-CNT/mwGO/RVC	7	3	50
mwGO/RVC	0	10	50
(b)			
9-CNT/mwGO/RVC			
1	9	1	10
2	9	1	30
3	9	1	50

In future discussions, these composite electrodes, depending on the ratio of a-SWCNT in the sample, will be identified as 9-CNT/mwGO/RVC (9 a-SWCNT: 1 mwGO-coated RVC electrode), 8-CNT/mwGO/RVC (8 a-SWCNT: 2 mwGO-coated RVC electrode), 7-CNT/mwGO/RVC (7 a-SWCNT: 3 mwGO-coated RVC electrode), CNT /RVC (10 a-SWCNT: 0 mwGO-coated RVC electrode), and mwGO/RVC (0 a-SWCNT: 10 mwGO-coated RVC electrode).

### Characterizations

#### Electrochemical Characterization

The capacitance of a-SWCNT, mwGO, and a-SWCNT/mwGO composite electrodes was measured using a cyclic voltammetry (CV). In this measurement, a-SWCNT/RVC, mwGO/RVC, or a-SWCNT/mw rGO/RVC electrode was taken as the working electrode (WE). The test was carried out in 1 M NaCl aqueous solution by scanning the voltage range − 0.2–1.0 V using a three-electrode system; RVC was taken as the counter electrode (CE) whereas Ag/AgCl (3 M NaCl) as the reference electrode (RE). Pt wire was used for making contact between WE and CE. The scanning rate for the measurement was taken as 5, 10, 20, 50, 100, and 200 mV/s. The electrochemical impedance spectra (EIS) for three electrodes namely mwGO/RVC, CNT/RVC, and 9-CNT/mwGO/RVC were measured over the frequency range 10^1^–10^7^ Hz. Galvanostatic charge-discharge (GCD) process was tested within voltage range 0–1 V.

#### Physical Characterization

Homogeneity and birefringence of the dispersion were checked by Olympus BH-2 microscope. The morphological test of the a-SWCNT-, mwGO-, and a-SWCNT/mwGO composite-coated RVC electrodes was done using a FESEM at voltage 1.0 kV. Furthermore, a-SWCNT and GO functionalization characterization was carried out by (1) visible absorption spectra, (2) Raman spectra (measured by a Raman spectrometer attached with a Raman microscope and CCD detector at the excitation wavelength of 632.81 nm where spectra were recorded over 30 s at 1.0 cm^−1^ resolution, and (3) XPS for determining the functional groups in carbon binding to carbon, and hydrogen and oxygen in GO and mwGO. The XPS binding energy spectra were obtained by passing energy 20 eV in the fixed analyser transmission mode. The XRD test was performed to determine the crystallinity and the interlayer distance between nanosheets. The XRD measurement was carried out using CuKα radiation (*λ* = 0.154) source that operated at 40 keV with a cathode current 20 mA. Movement, the thermal stability of the graphite, GO, and mwGO materials were determined by thermogravimetric analysis (TGA). The experiment was performed using a Q500 (TA Instruments) apparatus at a ramp rate of 5 °C/min in air, with a combined gas flow of 10 ml min^−1^ nitrogen (N_2_) and 90 ml min^−1^ air from 25 to 800 °C.

## Results and Discussion

### Chemical Conversion of Graphite to Graphene Oxide

To chemically convert the graphite into GO, the reaction of graphite with sulphuric acid (H_2_SO_4_) in the presence of KMnO_4_ is shown in Eq. (1). Graphite bisulfate (GB) (${\mathrm{C}}_{24}^{\mathrm{s}}{\mathrm{H}\mathrm{SO}}_4^{-}.2{\mathrm{H}}_2{\mathrm{SO}}_4\Big)$, was prepared using a chemical oxidative method by the intercalating graphite with a mixture of KMnO_4_ and concentrated sulphuric acid [57].

1 $$ 5{\mathrm{C}}_{24}+{\mathrm{KMnO}}_4+17{\mathrm{H}}_2{\mathrm{SO}}_4\to {\mathrm{C}}_{24}^{\mathrm{s}}{\mathrm{H}\mathrm{SO}}_4^{-}.2{\mathrm{H}}_2{\mathrm{SO}}_4+{\mathrm{MnSO}}_4+4{\mathrm{KHSO}}_4+{\mathrm{H}}_2\mathrm{O} $$

At the same time, rapid bubbling occurred because of releasing CO_2_ from the reaction mixture according to Eq. (2) [57]:

2 $$ 5\ \mathrm{C}+4{\mathrm{KMnO}}_4+8{\mathrm{H}}_2{\mathrm{SO}}_4\to 5\ {\mathrm{CO}}_2+4{\mathrm{MnSO}}_4+4{\mathrm{KHSO}}_4+6{\mathrm{H}}_2\mathrm{O} $$

The intercalated compound was hydrolyzed by adding water to form graphite oxide. The decomposition of permanganate ions into manganese (IV) ions was carried out by adding H_2_O_2_, and thereafter, the mixture was vacuum filtrated to remove manganese (IV) ions. The resultant was washed with HCl solution to ensure that no undesirable Mn(OH)_2_ was formed [25]. The inset in Additional file 1: Figure S4 (b) shows an optical image of graphene oxide after washing and vacuum drying, at the bottom of a glass vial.

### Surface Chemistry of GO

EDX spectra, FTIR spectra, and XRD patterns were used to confirm the complete oxidation of graphite flakes into GO using strong acid.

### Energy-Dispersive X-Ray Spectroscopy

Additional file 1: Figure S4 (a and b) shows the FESEM images and corresponding EDX spectra of graphite flake powder and as-prepared GO, respectively. The smooth leaf structure of graphite flakes was completely changed into the uniform rough and porous structure containing valleys and elevated regions which reflect vast amounts of sheet stacking after the chemical oxidation process, which is clearly viewed from the SEM images of graphite and as-prepared GO given as insets in Additional file 1: Figure S4 (a) and (b), respectively. The crystal structures of graphite flake powder and as-prepared GO are identified using XRD patterns (Additional file 1: Figure S4 (c)). A strong and sharp peak was observed for graphite flakes at 26.6° corresponding to a basal spacing *d*_002_ = 0.343 nm. This indicated a highly ordered structure of graphite. The XRD pattern of GO, on the contrary, exhibited at low-angle region (10.8°, 001 reflection) corresponded to a basal spacing of *d*_001_ = 0.820 nm, whereas the broader peak at 2*θ* = 20.7° corresponded to the (*d*_002_ = 0.435 nm) crystal plane of graphite and amorphous carbon [6, 12]. The observed results are in good agreement with the other GO precursors [6, 52]. This is because, during oxidation, the oxygen-containing functional groups are introduced into the carbon lattice [64]. Hence, the results indicated that the interlayer spacing of GO (*d*_002_ = 0.435 nm) was increased because of the oxidation of graphite flakes (*d* spacing = 0.343 nm). In addition to SEM images and XRD, the corresponding EDX spectra in Additional file 1: Figure S4 (b, for GO) reveal the formation of a new structure with a higher oxygen content of 30.1 wt% O (the oxygen content of graphite is too low to be detected (Additional file 1: Figure S4 (a)) and lower C/O ratio of 2.3; indicating the oxidation of graphite flakes and formation of GO. The higher oxygen content of GO compared to the graphite is because of the formation of epoxide, carboxylic acid and –OH groups on the edges and the basal planes of the carbon structure, respectively [16]. Besides C and O atoms in Additional file 1: Figure S4 (b), no peaks existed in the EDX spectra of GO; which indicates that no impurities, presence of sulphur and manganese, have been left as residuals of H_2_SO_4_ and KMnO_4_ used in the preparation procedure.

### Fourier Transform Infrared Spectroscopy

The presence of oxygen-containing functional groups was evident from the Fourier transform infrared (FTIR) spectrum as shown in Additional file 1: Figure S4 (d). This confirmed that the graphite flakes were successfully oxidized to the graphene oxide. The broad and wide peak observed at 3386 cm^−1^ correspond to the –OH stretching vibrations of the hydroxyl (C–OH) groups and water, and the epoxy (C–O–C) group peak at 1228 cm^−1^ [33, 41, 74]. These groups usually are present throughout the basal planes of GO [10]. In addition, the sharp intense peak at 1587 cm^−1^ is ascribed to (C=C) benzene rings, and the absorption bands at 1720 and 1047 cm^−1^ can be ascribed to the stretching vibration of carboxyl (C=O) and alkoxy (C–O) groups [37, 41, 54, 69], respectively, which usually were distributed at the edges of these 2D nanostructures [10].

### Exfoliation and Reduction of GO Through Microwave Irradiation

Microwave irradiation technique was used for preparing exfoliated graphite (EG) [17, 58] and GO [6]. In addition, microwave-assisted reduction of GO can be achieved in less than 1 min by the treatment of GO precursor within a microwave oven [6, 78]. Therefore, the microwave irradiation method was used to exfoliate and reduce graphene oxide. The GO powders were irradiated within a microwave oven for 10 s that largely expanded its volume and fumed violently. The SEM image of GO and mwGO films are shown in Additional file 1: Figure S5 (a) and 2 (b), respectively. The image in Additional file 1: Figure S5 (b) shows that the graphene sheets expanded, developed an accordion-type structure of highly 3D macropores folded with each other, and formed an interconnected network with minimal re-stacking compared with GO before microwave irradiation in Additional file 1: Figure S5 (a) [6, 58] which were transparent to the electron beam and ion salts. In addition, the SWCNT can be sandwiched into the layers more easily [77]. Furthermore, the XRD spectra, Additional file 1: Figure S5 (c), shows one distinct broad peak for mwGO at 2*θ* = 20.7° corresponding to the (*d*_002_ = 0.435 nm) crystal plane of graphite, and the sharp peak at 2θ = 10.8° is heavily suppressed because of the rapid expansion of GO layer. This result is in good agreement with other mwGO precursors [6] and corresponding to the removal of some of the functional groups of GO and successful exfoliation of graphene [18, 64, 72]. Besides XRD and SEM images, the corresponding EDX spectra, presented in Additional file 1: Figure S5 (d) and S5 (e), revealed the formation of a new structure with the lower oxygen content of 9.9 wt% O and higher C/O ratio of 9.1. This indicated that reduced graphene oxide (rGO) was formed after irradiation. The lower oxygen content of rGO compared to GO was because of releasing epoxide, carboxylic acid, and –OH groups on the edges and the basal planes of the carbon structure, respectively. It can be viewed from the inset in Additional file 1: Figure S5 (e) that there is a dramatic expansion of the GO powder (placed inside the glass vial (inset Additional file 1: Figure S5 (d)) that yielded a black and fluffy mwGO powder. Zhu et al. demonstrated that further treating mwGO powder with microwave irradiation-induced sparking and even burnt the sample [78].

### Surface Chemistry of GO After Microwave Irradiation

The chemical changes that were observed in GO because of microwave irradiation were investigated by Raman spectra, TGA, XPS, and XRD spectra to confirm its reduction and exfoliation.

### Raman Spectra

Raman spectroscopy is a powerful diagnostic tool used for the structural and qualitative characterization of carbons [14, 15], particularly for investigating the presence of any defect and ordered or disordered structure of graphite and graphene. Many scientific papers are mentioning that there are three typical peaks always attributed to D, G, and 2D bands of graphite, GO, and rGO that are observed at about 1350, 1580, and 2680 cm^−1^, respectively [42, 55]. The 2D band is always a strong band in graphene even when there is no D band present, and it does not represent defects. Additional file 1: Figure S6 exhibits the Raman spectral curves of natural graphite flakes, as-prepared GO, and GO after microwave irradiation. It is clearly viewed from the Raman spectra that graphite exhibits two peaks at G and 2D bands. The chemical oxidation on graphite to produce graphene oxide (GO) resulted in the appearance of a D band at a lower wave number compared to the G band coupled with the disappearance of the 2D band peak. The D band peak of GO is associated with the breathing modes of rings or *κ*-point phonons of A_1g_ symmetry of graphene oxide occurring at 1321 cm^−1^, while the G band peak, associated with first-order scattering of the stretching vibration mode E_2g_ observed for carbon sp^2^ atoms domains, occurs at 1587 cm^−1^ [67]. The integrated intensity ratio (*I*_D_/*I*_G_) of these two Raman bands has been demonstrated to depend upon the physical state of the graphitic carbon and can reveal the ordered and disordered crystal structures of graphene. As expected, after microwave irradiation, both D and G band peaks were slightly shifted to 1330 cm^−1^ and 1590 cm^−1^, respectively, and the ratio of the *I*_D_/*I*_G_ band decreased from 1.26 to 1.08. This decrease suggests a slight reduction of the GO [29, 65]. Moreover, this microwave-assisted reduction of GO has a good advantage over chemical reduction by ultrasonic dispersion or rapid thermal expansion followed by chemical reduction because chemical reduction often involves highly toxic chemicals, require long reduction time, or require high-temperature treatment [20, 53]. 

### XPS Spectra Analysis

XPS was employed for investigating the surface chemical states of GO and microwave-irradiated graphene oxide (mwGO) powder by observing any change in the binding energy of C1s peaks. Additional file 1: Figure S7 depicts the XPS spectra of GO and mwGO within the binding energies scanned range from 280 to 294 eV, which presents information on the main chemical components in the samples from the detected peaks (C1s spectra) of both samples. It can be seen that in Additional file 1: Figure S7 (a), the C1s XPS spectrum of GO clearly indicates a considerable degree of oxidation with three components corresponding to carbon atoms in different functional groups [68]: the non-oxygenated C at 284.4 eV corresponds to C1s of sp^2^ (C=C), the carbonyl carbon (C=O) at 287.2 eV, and the carboxylate carbon (O–C=O) at 288.4 eV. Based on the XPS analysis, the as-prepared GO had an atomic ratio for carbon/oxygen of 2.91. After microwave irradiation of GO (Additional file 1: Figure S7 (b)), the number of C=C band increased from 34.21 to 56.47%, revealing that the graphitic structure of rGO is remarkably restored, while the C/O atomic ratio of mwGO was found to be 5.32. The XPS results show significant removal of the oxygen-containing groups because of exposure to the microwave radiation as reported for graphite oxide and graphene oxide [6, 33, 78]. It can also be mentioned that the slight shifting of the de-convoluted peaks for mwGO is because of the enhanced regular structure compared to the GO [40].

### Thermogravimetric Analysis

Thermogravimetric analysis (TGA) was used for studying the thermal stability of natural graphite flakes, GO, and microwave-irradiated graphene oxide (Additional file 1: Figure S8). The TGA curves represented the mass loss during the heating in air. It is clear that pristine graphite is thermally very stable, only 4.8 wt% mass loss occurred upon heating to 600 °C as in agreement with the Song et al.’s report [56]. For graphene oxide (GO), three stages of weight loss are apparent, as shown in Additional file 1: Figure S8. The first one is below 120 °C, corresponding to the loss of intercalated water that was not removed from drying. The second one, between 120 and 300 °C, corresponding to the removal of oxygen-containing functional groups, such as C–O, C=O, and O–C=O [27, 40, 64]. This major weight loss is related to degradation and decomposition, whereas the third weight loss that occurs at approximately 530 °C is because of the bulk pyrolysis of the carbon skeleton [62, 64]. This pyrolysis is also observed in mwGO at 565 °C and in graphite at 680 °C. The TGA analysis indicates the incomplete reduction of the functional groups by microwave because roughly 15 wt% of oxygen-containing groups did not undergo reduction.

### Dispersion of mwGO in DMF

Recently, sonication has been extensively used as an exfoliation and dispersion strategy for producing graphene nanosheets in a liquid medium [24]. The first parameter to be investigated was the impact of sonication time for dispersing mwGO in DMF. The prepared dispersion contained mwGO 0.1% w/v in 15 ml DMF; this is one of the best solvents reported for GO dispersions because it gave the highest spacing between GO sheets of up to 1.05 nm and the smallest GO sheet thickness, down to 0.83 nm [26]. It is necessary to determine the optimum sonication time for dispersing mwGO in DMF because excessive sonication may result in the shortening or creating a defect in the sheets. The dispersion and stabilization of mwGO were performed using UV-VIS spectroscopy. The visible spectra (between 300 and 1100 nm) of mwGO dispersion with respect to sonication time are presented in Additional file 1: Figure S9 (a). All mwGO solutions were diluted five times by DMF before measuring their visible spectra, using 1-cm path length quartz cuvettes and considering the baseline for DMF. The absorbance of the supernatant at 660 nm was considered for plotting with respect to sonication time and represented in Additional file 1: Figure S9 (b). Peak intensities were read directly from the as-measured absorption spectrum. It is observed that, with the increase in sonication time, the mwGO absorption bands became more pronounced, indicating that the good dispersion of mwGO happened with respect to time. Moreover, there is very little change in the absorption spectra of the dispersion after further sonicating beyond 35 min is observed. This implied that the solvent was saturated and the minimum time for effective dispersion of the mwGO was 35 min. The photographs of mwGO dispersions before starting the sonication and after 35 min of sonication are shown in the inset photographs in Additional file 1: Figure S9 (a). It is observed that with the increase in sonication time, the colour of the dispersion is becoming gradually black, indicating that the mwGO was homogenously dispersed within DMF. It is well known that the complete dispersion is affected by sonication energy. The visible absorbance of mwGO dispersion with respect to sonication energy is presented in Additional file 1: Figure S9 (c), which shows the sonication energy required to disperse 15 mg of mwGO in 15 ml DMF. It is obvious that the energy increased with sonication time. The energy was 12 kJ at 2 min and was 90 kJ at 15 min. The effective dispersion of mwGO was achieved at the minimum energy of 210 kJ at 30 min because the dispersion’s absorbance remained constant beyond the sonication energy of 210 kJ. This energy, as represented in Additional file 1: Figure S9 (c), was for 35 mg in DMF (0.1% w/v). This indicates that 14 kJ energy was required per milligram of mwGO in a 0.1% w/v solution.

### Electrodes Surface Morphology

**Fig. 1:**
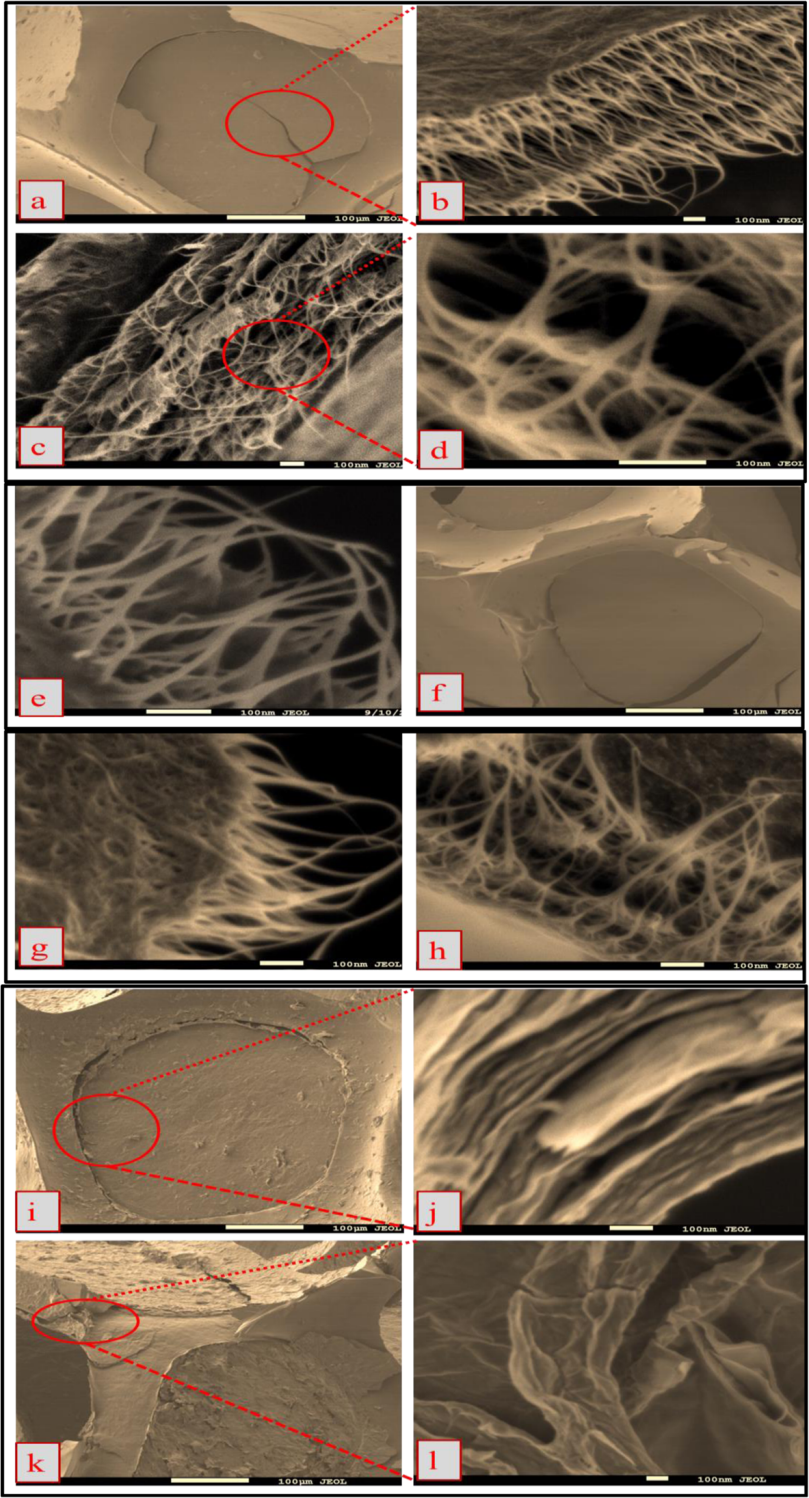
SEM images of the **a** top surface, **b** 45° view, and **c**–**e** cross-section of 9-CNT/mwGO/RVC electrode; **f** top surface, **g** 35° view, and **h** cross-section of 8-CNT/mwGO/RVC electrode; **i** top surface and **j** 45° view of 7-CNT/mwGO/RVC electrode; and **k** top surface and **l** 45° view of mwGO electrode

Figure 1 shows the surface morphology of the 9-CNT/mwGO/RVC, 8-CNT/mwGO/RVC, 7-CNT/mwGO/RVC, and mwGO/RVC electrodes using SEM. The deposition in all the composite electrodes is clearly visible and is homogeneously distributed on the surface and pores of the RVC substrate as presented in Fig. 1a, f, i. The representative SEM image which was taken from a 45° view of the 9-CNT/mwGO/RVC electrode in Fig. 1b showed that the top surface of the composite material coating appeared like a textile because the CNTs and mwGO were regularly distributed on the RVC electrode and its pores, being partly parallel and partly perpendicular to the surface. In addition, CNTs are distributed in a highly tangled fashion with one another and systematically distributed like a web, showing the obvious domination of CNTs within the composites and with the CNTs uniformly wrapped in between the curled graphene sheets. The cross-section of the electrode shown in Fig. 1c indicates a layered structure, and the CNTs are uniformly sandwiched between the GO sheets making a multilayered 3D network structure having highly porous architecture within the electrode where graphene nanosheets conductively bridge the pores between the CNT particles. Moreover, it is revealed that there are even distributions of CNTs and graphene sheets throughout the resultant structure. It is clearly apparent that many gaps that interface between graphene sheets and CNTs are formed as an open pore system (Fig. 1d), are favourable for easy electrolyte ion access to the surface of mwGO and CNTs to form electrical double layers [39], and may increase the surface area [50]. This is because of the enhanced van der Waals force of attraction and friction, which is in good agreement with the published reported results [73, 75, 76]. In addition, it is observed in Fig. 1d that the voids or “pores” present between the CNT Matts are in the micron level through which the ions can free diffuse thus enhancing electrosorption. The CNT nano-networks have acted as useful nano-spacers to diminish the face-to-face aggregation of CNTs. This 3D porous structures have exposed extensive surface areas that facilitate for diffusing ions through these pores and perform a higher level of electrosorption [60]. Moreover, the strands of CNTs adhere to each other to form smaller aggregation bundles (10–30 nm) compared with usual CNT diameters (50–80 nm) [49] and bundles of CNTs in CNT/RVC electrode (25–50 nm). This decrease in aggregation bundles of CNTs is expected when combining graphene sheets with CNTs according to the data reported by Zhang et al. [72]. The tightly wrapped nanotube bundles lead to a reduced electrode resistance [23] and maximize the surface area that eventually allows large capacitance value [45]. These result in increased ion capture and conductivity of 9-CNT/mwGO/RVC electrode.

The top view of 8-CNT/mwGO composite materials (Fig. 1g) obviously shows that nanotube bundles are still dominant and highly tangled, wrapped inside the composite. The strands of CNTs adhere to form bigger aggregation bundles compared with 9-CNT/mwGO composite materials. The cross-section of this composite electrode shown in Fig. 1h indicates a layered structure and the CNTs are uniformly sandwiched between the GO sheets making a multilayered 3D network structure with good porous architecture inside it. Obviously, the graphene nanosheets restacking and gaps interfacing between graphene sheets and CNTs to form a favourable open pore system was decreased. This results in difficult electrolyte ion access to the surface and minimizes surface area compared to 9-CNT/mwGO composite material. This phenomenon could possibly because of the minimization of surface area and specific capacitance. It is evident that, in Fig. 1j, when the mwGO ratio reaches to 3 in the composite, the graphene nanosheets dominate in the composite and the cross-section surface was completely changed into the 3D uniform rough multilayer and porous structure containing voids with CNTs distributed in them but is difficult to clearly image. This structure results in the reduction of electrolyte accessibility to the surface area. In contrast, Fig. 1k exhibits the top surface of mwGO/RVC electrode. The top surface of mwGO has rippled and crumpled structures and wrinkled structures with clear aggregation, and the stacked structure of the nanosheets in the cross-section image of mwGO (Fig. 1l) can be clearly viewed. The cross-section SEM reveals a curled morphology consisting of rippled structures. Consequently, graphene sheets with curled or wrinkled morphologies will present the greatest difficulty for accessing electrolyte to the surface area. Therefore, the specific capacitance in mwGO/RVC electrode is expected to be the smallest, comparing amongst all composite electrodes.

### Electrochemical Behaviour of Electrodes Studied Using CV

The electrochemical properties of all the coated RVC electrodes were measured using CV. In this article, CV was used to measure the variation of capacitance of the electrodes with respect to loading level and scan rate and to check the electrode cyclic stability using 1 M NaCl solution within the voltage range between − 0.2 and 1.0 V vs Ag/AgCl in a three-electrode system. A RVC was taken as the counter electrode whereas mwGO/RVC, CNT/RVC, 7-CNT/mwGO/RVC, 8-CNT/mwGO/RVC, and 9-CNT/mwGO/RVC as the working electrodes.

### Capacitive Behaviours of mwGO/RVC, CNT/RVC, and Various CNT/mwGO/RVC Composite Electrodes

**Fig. 2:**
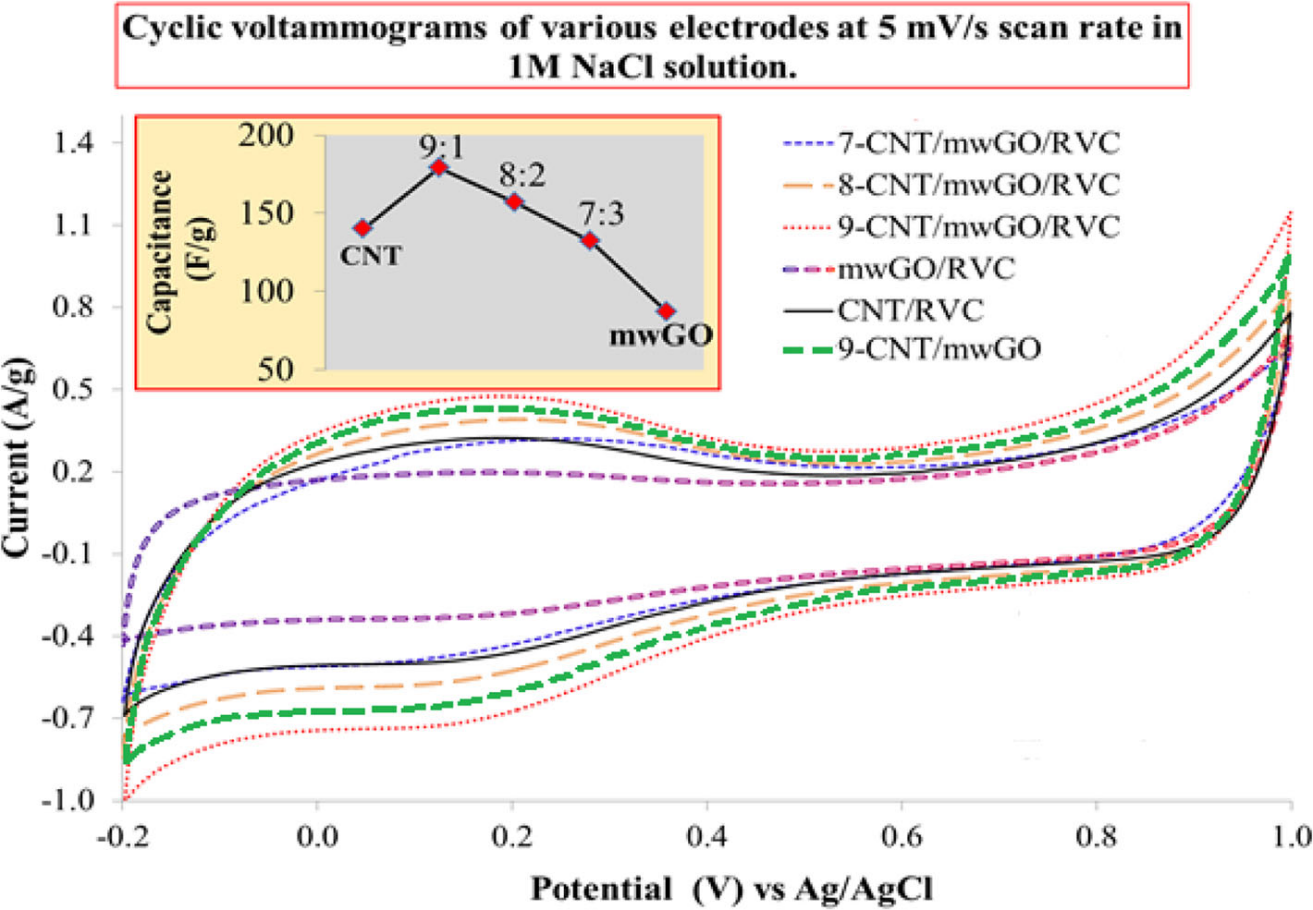
Comparison of CVs and specific capacitance (inset) for mwGO/RVC, CNT/RVC, 9-CNT/mwGO, 7-CNT/mwGO/RVC, 8-CNT/mwGO/RVC, and 9-CNT/mwGO/RVC

It is well known that the electrochemical capacitance is one of the common factors to govern the capacitance. The electrodes’ capacitive behaviour is contributed mostly by the formation of charge at the electrochemical double layer and negligibly by the pseudo-capacitance. The capacitance value of all materials coated RVC electrodes was calculated by integrating the curve area of the CV curve. Figure 2 presents the CV curves of CNT/mwGO composite without RVC, and mwGO, CNT, and various ratios of mwGO in CNT/mwGO composite materials coated RVC electrodes measured at scan rate 5 mV/s and presented with respect to current per gram of materials. For all samples, the CV curves have one pair of redox peaks that observed within the potential range − 0.2 to 0.4 V, probably owing to a small quantity of oxygen-containing functional groups in all electrodes [71]. In addition, the CV curves are looking like distorted quasi-rectangular, indicating its pseudo-capacitive nature with no significant Faradaic reaction being observed for any electrode. This indicates that ions are adsorbed on the electrode surface by forming an electric double layer due to Coulombic interaction rather than electrochemical reaction [31]. It is worth noting that in Fig. 2, the current density of mwGO is much lower compared to CNT and CNT/mwGO. Therefore, mwGO-coated RVC electrode shows the lowest specific capacitance (87.21 F/g) value compared to other electrodes. This specific capacitance value is very small compared to other studies for rGO at the same experimental condition (140 F/g) [32, 63]. The low specific capacitance of mwGO indicates low exposure of surface area since they are proportionally related to each other. This experimental result could be ascribed to the fact that the graphene sheets are loosely stacked or folded with each other to construct interconnected 3D macropores in mwGO/RVC electrode as shown in SEM image (Additional file 1: Figure S3) that leads to formation of aggregates, and the distance between interlayers of sheets (0.435 nm) is too small to allow the hydrated sodium ions (radius = 0.358 nm) access. This results in the poor exposure of the surface area to form the electrical double layer with electrolytes. Therefore, the mwGO agglomerates show comparatively low specific capacitance.

We reported the CV curve of the CNT/RVC electrode, where the value of specific capacitance measured at 5 mV/s scan rate was 139.65 F/g [2]. This capacitance is increasing when the mwGO material is combined with CNT material at the approximate ratio of 2:10, as presented in Fig. 2. The inset in Fig. 2 exhibits that the 9-CNT/mwGO/RVC electrode possesses the highest specific capacitance value (179.39 F/g), which is 29% higher compared to the CNT/RVC electrode. This enhancement is due to the synergistic effect of CNT and mwGO. The incorporation of mwGO into CNT forms an advantageous network structure that conductively bridges the pores between the CNT particles by facilitating the rapid electrolytic ions’ transport inside the electrode materials, and thereby, the electrode’s surface area is increased. This surface area increased due to the large external active surface area of mwGO sheets and the diameter of bundling of CNTs in CNT/mwGO/RVC composite electrode being smaller than 25 nm. This is smaller compared to the diameter of bundling of CNTs alone, reported elsewhere [2]. The specific capacitance value, measured at 5 mV/s scan rate for 9-CNT/mwGO electrode, was 167.56 F/g, less compared to 9-CNT/mwGO/RVC composite electrode (179.39 F/g). This high value of specific capacitance for 9-CNT/mwGO/RVC electrode may be due to amazing property of RVC, which reduce the resistance of solution flow through the electrode thereby increasing the possibility of ions to reach all electrode surfaces.

The inset in Fig. 2 exhibits a decreasing trend of the specific capacitance value with further increase in mwGO content in the CNT/mwGO/RVC composite electrode. For example, the specific capacitance values of 8-CNT/mwGO/RVC and 7-CNT/mwGO/RVC electrodes are found to be 156.47 and 132.60 F/g, respectively. This decrement in specific capacitance value is because of the low specific surface area of mwGO. Furthermore, this decrement in specific capacitance value can be ascribed to the difficulty of electrolytic ions’ transport into the porous CNT structure because the excessive mwGO mostly covered its surface. Interestingly, the CNT/RVC electrode shows a higher value of specific capacitance compared to 7-CNT/mwGO/RVC electrodes. From the above-going discussion of specific capacitance, it can be suggested that the electrosorption capacity performance of 9-CNT/mwGO/RVC electrode will be better compared to all other electrodes. Moreover, the specific capacitance result of mwGO/RVC electrode, being lower compared to other electrodes, suggests not to applying this electrode in the CDI system selected for experiments because the electrosorption capacity will be very small compared with CNT/RVC and CNT/mwGO/RVC electrodes.

### The Effect of Increasing Loading Level on Capacitive Behaviours

In the previous section, it is reported that the 9-CNT/mwGO/RVC composite electrode has shown the highest specific capacitance value. Hence, this electrode has been selected for studying the effect of increasing loading level of composite materials in geometric area terms and geometric volume terms on its capacitive behaviours. The capacitive behaviours of all 9-CNT/mwGO/RVC electrodes, which had similar geometric volume (2.16 cm^3^) of RVC electrodes with various amounts of composite materials coated, 10, 30, and 50 mg, were studied by CV. The maximum loading level was set at 50 mg, because beyond this level, the pores of the RVC electrode was completely filled, which is evident from the top view of SEM image (Fig. 1a).

**Fig. 3:**
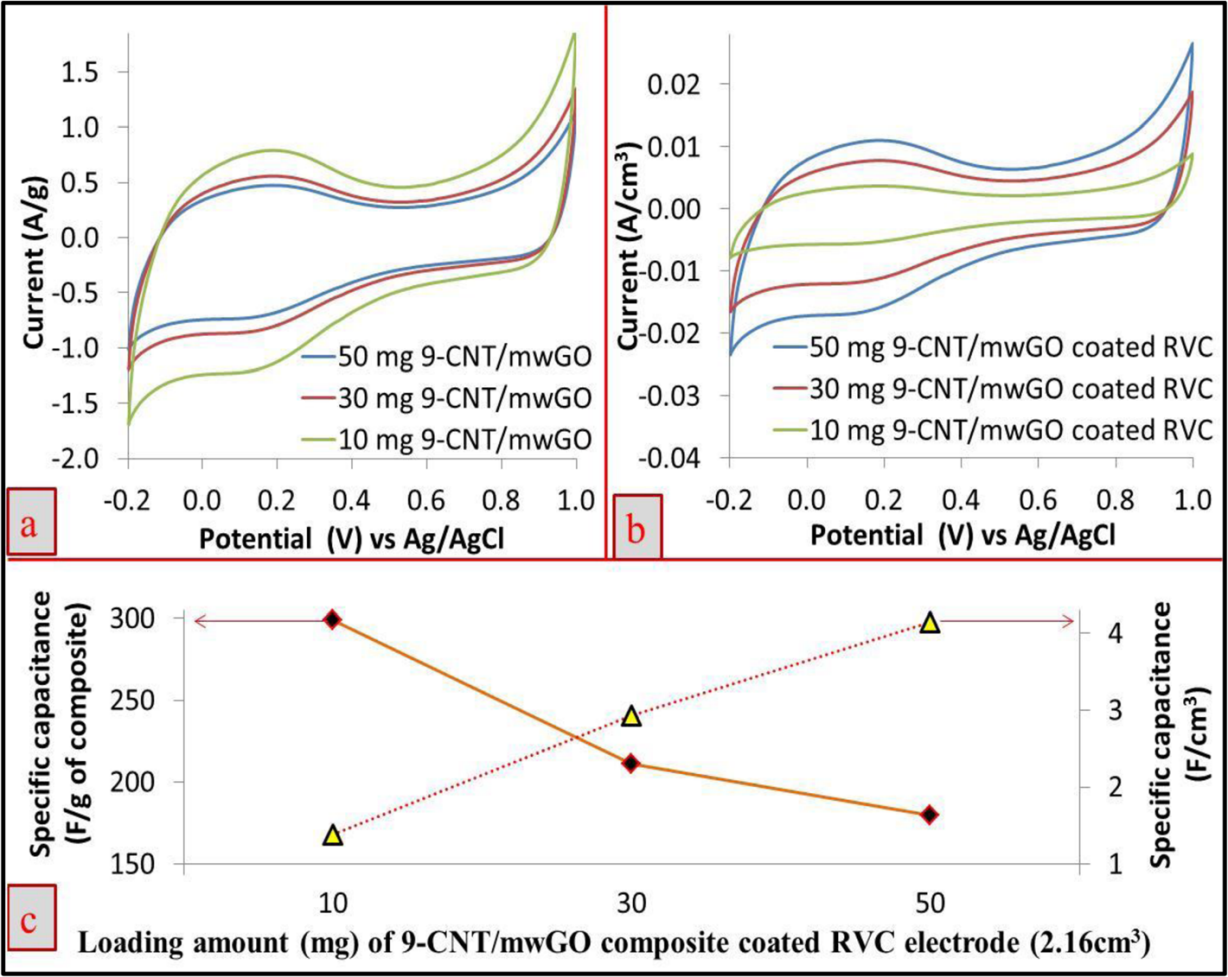
CV of 9-CNT/mwGO composite-coated RVC electrodes in terms of **a** current per gram of composite and **b** current per geometric volume of electrode. **c** Capacitance of the electrodes per gram of composite and per geometric volume of the electrode

Figure 3a, b shows the CV curves (measured at 5 mV/s) of different 9-CNT/mwGO-loaded RVC composite electrodes with respect to current per gram and current per geometric volume of electrode, respectively. As expected, the CV behaviours of electrodes are keeping with earlier results reported for the CNT electrodes [2, 4] that is with the increase in loading of composite material within the RVC electrode resulted in the decrement of current per gram of 9-CNT/mwGO composite (Fig. 3a) and increment in the current per geometric volume of electrode (Fig. 3b). The capacitance results per unit gram of composite material and per unit geometric volume of electrode are shown in Fig. 3c as F/g and F/cm^3^, respectively. Fig. 3c shows that the increase in loading of 9-CNT/mwGO composite material in the RVC electrode resulted in the decrease of capacitance value of 9-CNT/mwGO composite material. For example, the highest and the lowest specific capacitance values for the 10 mg and 50 mg composite material loaded were 298.98 F/g and 179.39 F/g, respectively. The capacitance value at 10 mg loading (298.98 F/g) of 9-CNT/mwGO/RVC electrode is higher compared to the previously reported value, 220 F/g [63]. On the contrary, it is also seen from Fig. 3c that the capacitance values, calculated with respect to geometric volume for 10, 30, and 50 mg 9-CNT/mwGO composite coated on the RVC electrodes was 1.39 F/cm^3^, 2.93 F/cm^3^, and 4.13 F/cm^3^, respectively. This indicates that the capacitance value increases with the increase in composite loading on RVC electrode. Furthermore, the calculated capacitance values with respect to geometric area (F/cm^2^) for the RVC electrodes coated with 10, 30, and 50 mg 9-CNT/mwGO composite material were 0.17 F/cm^2^, 0.36 F/cm^2^, and 0.50 F/cm^2^, respectively, indicating that the capacitance value has increased with the increase in coating amount of composite material on RVC electrodes. This may be due to the decrease in porosity of the electrode because of composite coating, which in turn results in the increase in its surface area. Hence, it is generalized from the above-going discussions that the value of specific capacitance with respect to both geometric area and volume has increased with the increase in amounts of material on the RVC electrode. However, the capacitances are significantly higher when expressed in terms of geometric volume.

### Effect of Different Scan Rates on the Electrode Capacitance

**Fig. 4:**
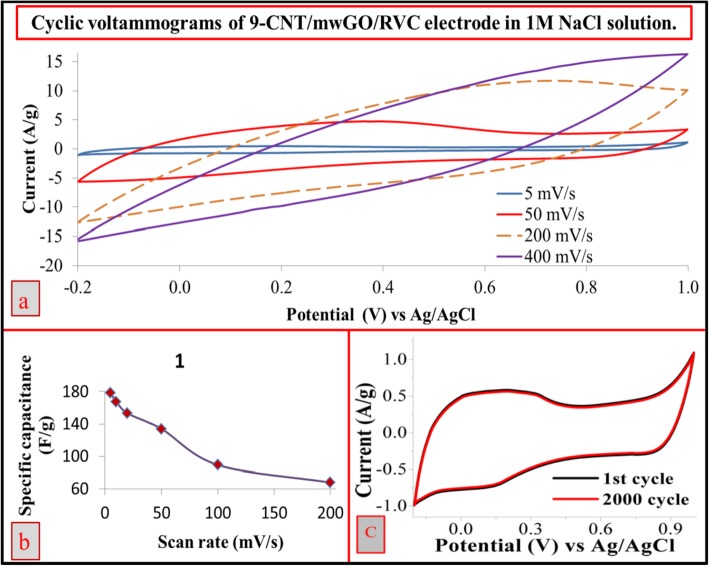
**a** CV and **b** specific capacitance at various scan rates and **c** the cyclic stability scanned at 20 mV/s using 9-CNT/mwGO/RVC electrode

The capacitance value of electrode with respect to increasing scan rate was performed in aqueous NaCl solution. Figure 4 represents the CV curve of 9-CNT/mwGO/RVC electrode with respect to different scan rate. The observed nearly rectangular shape of CV curves at the scan rates 5 and 50 mV/s (Fig. 4a) is suggesting the formation of high electrochemical double-layer capacitors (EDLC) [11]. CV study shows that the carboxyl and carbonyl groups contributed to the increase in capacitance value of CNTs that are pseudo-capacitive in nature [35, 46]. This suggests the existence of an intimate contact between the electrolyte and the active material, which in turn has resulted in excellent charge transfer kinetics because of the formation of porous and cross-linked structure. In can be clearly mentioned that the polarization that arises due to high scan rate has simultaneously shifted the anodic and anodic peaks toward high and negative potential, respectively [35]. However, further increase in scan rate beyond 50 mV/s (Fig. 4a) has resulted non-rectangular shaped curves, which indicates resistance-like electrochemical behaviour, because the electrode is very porous which hinders the migration of NaCl to the pores and this becomes pronounced when the scan rates of CV are increased [35]. This is why the capacitance value of electrodes is continuously decreased with the increase in scan rate (Fig. 4b). This feature can be ascribed to the resistance of the electrolyte and the inner resistance of ion diffusion with certain carbon micro-pores whose surface is only partially accessible to electrolytes. This becomes pronounce under comparatively high scan rates because of the differential depletion of the electrolyte concentration [43]. It is observed that there is a sharp decrement in the specific capacitance value of electrode as the scan rate increases beyond 50 mV/s. As expected, the capacitive volume should be increased with the increase in the scan rate because it has been reported for all cases of CNT and graphene composite [34, 35, 59]. Furthermore, the cyclic stability test for 9-CNT/mwGO/RVC electrode was carried out and presented in Fig. 4c. The CV curve showed very high stability, 97% stable after 2000 cycles of test. In addition, Table 2 shows the specific capacitance for all electrodes with respect to F/g of material, F/cm^2^ geometric area of the electrode, and F/cm^3^ geometric volume of the electrode, in 1 M NaCl solution at various scan rates. It is observed in the table that the electrodes show high capacitance value when scanned at a low rate and low capacitance value when scanned at a high rate. For instance, the specific capacitance of 9-CNT/mwGO/RVC electrode was 179.39 F/g (0.50 F/cm^2^ or 4.13 F/cm^3^) and 67.79 F/g (0.19 F/cm^2^ or 1.57 F/cm^3^) at 5 and 200 mV/s scan rates, respectively. It is apparent in Table 2 that the behaviour of specific capacitance with respect to geometric volume and geometric area followed the specific capacitance in terms of the mass of materials with respect to scan rate. This was expected because the mass of materials was similar in all electrodes (50 mg). It is interesting to note that the specific capacitance per geometric area of the mwGO-coated RVC electrode at 50 mV/s scan rate was 0.17 F/cm^2^. This specific capacitance increased around nine times compared with the same mwGO prepared as buckypaper (0.019 F/cm^2^), tested under the same conditions using 1 M NaNO_3_ solution [6].

**Table 2:** Specific capacitance value of different electrodes measured with respect to mass (F/g), area (F/cm^2^), and volume (F/cm^3^)

Scan rate (mV/s)		5	10	20	50	100	200
Sample							
mwGO/RVC							
Specific capacitance	F/g	87.21	75.87	69.77	61.05	47.97	34.88
	F/cm^2^	0.24	0.21	0.19	0.17	0.13	0.10
	F/cm^3^	2.02	1.75	1.61	1.41	1.11	0.81
CNT/RVC							
Specific capacitance	F/g	139.65	131.27	117.70	103.34	68.43	51.67
	F/cm^2^	0.39	0.37	0.33	0.29	0.19	0.14
	F/cm^3^	3.23	3.04	2.75	2.39	1.58	1.20
9-CNT/mwGO/RVC							
Specific capacitance	F/g	179.39	167.69	153.42	133.79	89.20	67.79
	F/cm^2^	0.50	0.47	0.43	0.37	0.25	0.19
	F/cm^3^	4.13	3.88	3.55	3.09	2.06	1.57
8-CNT/mwGO/RVC							
Specific capacitance	F/g	156.47	143.95	131.43	112.66	73.54	57.89
	F/cm^2^	0.44	0.40	0.37	0.31	0.21	0.16
	F/cm^3^	3.62	3.33	3.04	2.61	1.70	1.34
7-CNT/mwGO/RVC							
Specific capacitance	F/g	132.60	112.71	106.08	92.82	59.67	39.78
	F/cm^2^	0.37	0.31	0.30	0.26	0.17	0.11
	F/cm^3^	3.07	2.61	2.45	2.15	1.38	0.92
9-CNT/mwGO							
Specific capacitance	F/g	167.56	154.85	141.72	122.38	81.46	62.24
	F/cm^2^	0.47	0.43	0.39	0.34	0.23	0.18
	F/cm^3^	3.78	3.52	3.28	2.82	1.87	1.43

It is important to note that the capacitance of the RVC electrode per geometric volume under the same conditions at a scan rate of 20 mV/s was discussed in the literature [2]. The RVC was 0.002 F/cm^3^, and the capacitance had increased by a factor of 1060 when the same electrode was filled completely by functionalized CNT material. It can be observed in Table 2 that the capacitance of the RVC electrode had increased by a factor of 1775 when filled completely with 9-CNT/mwGO material. This is related to the large surface area of 9-CNT/mwGO/RVC compared to a RVC electrode according to the Randles-Sevcik relationship [7].

### Electrostatic Impedance Spectra

**Fig. 5:**
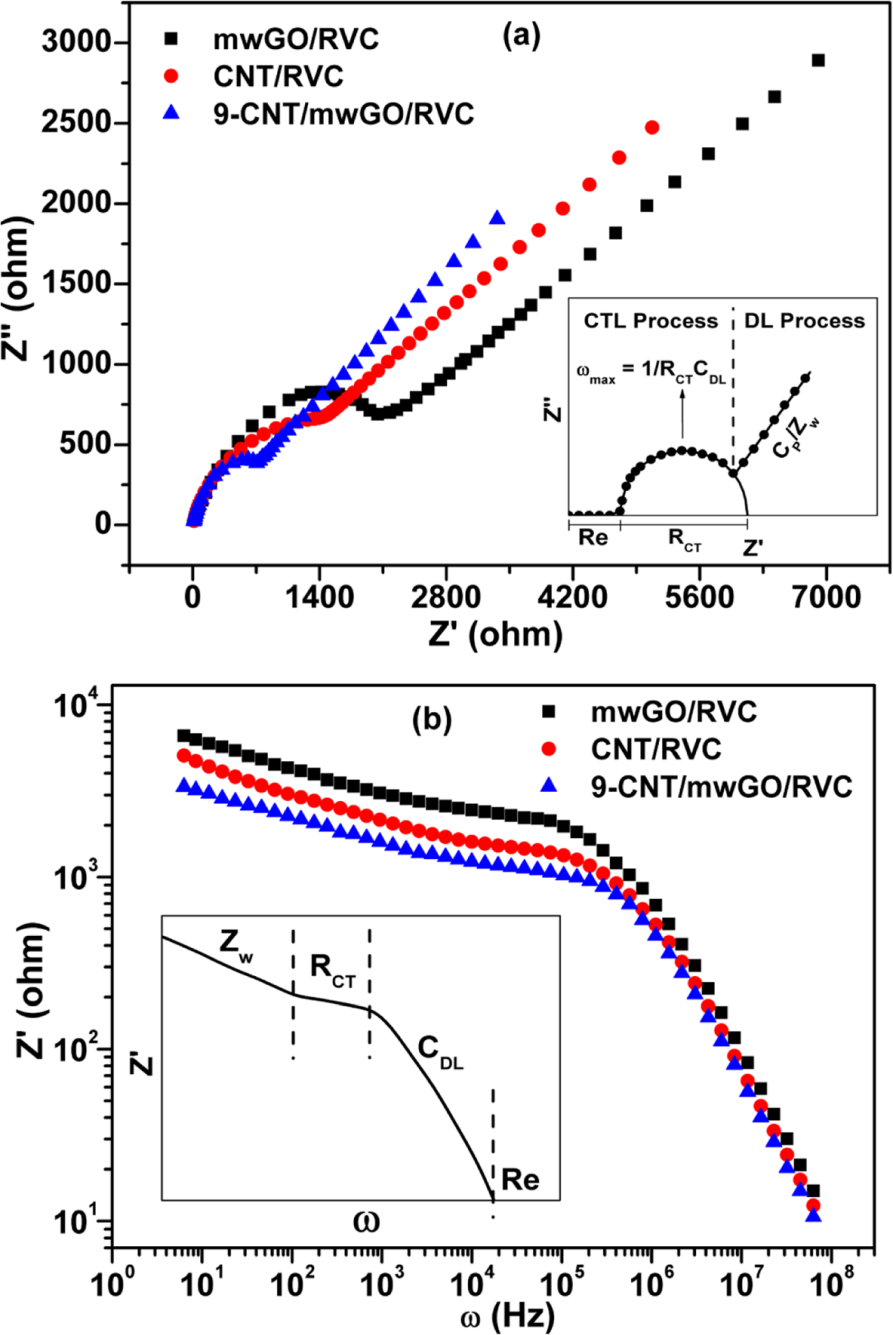
**a** Nyquist plot (inset diagram) and **b** Bode plot of mwGO/RVC, CNT/RVC, and 9-CNT/mwGO/RVC composite electrodes

The EIS of mwGO/RVC, CNT/RVC, and 9-CNT/mwGO/RVC electrodes are shown in Fig. 5. The plot of the real part of impedance (*Z*′) against the imaginary part impedance (*Z*″) is known as the Nyquist plot, which is represented in Fig. 5a for all three electrodes. It is observed from this figure that all the electrodes exhibit semicircle over high-frequency region and straight spike over the low-frequency region. The observance of semicircle over the high-frequency region indicates a charge-transfer limited (CTL) process, which is also known as redox process. Moreover, the observance of a straight spike implies a diffusion-limited (DL) process. Both processes are shown diagrammatically in the inset of Fig. 5a for better understanding. The appearance of semicircle over the high-frequency region is because of electrode polarization. The diameter of semicircle is accounting for polarization/charge-transfer resistance, which is concerning the ion diffusion through the macro-porous structure of the electrode. The figure shows that the diameter of semicircle is reducing when we proceed according to the order mwGO/RVC > CNT/RVC > 9-CNT/mwGO/RVC. This implies that the resistive behaviour of the electrodes also follows the same trend, but the ionic infiltration/charge-transfer on the surface of electrode follows the reverse trend. It is shown in the inset of Fig. 5a that the intersection of the semicircle with the *x*-axis at high frequency represents electrolytic resistance (Re) and at middle frequency represents charge-transfer resistance (*R*_CT_). Hence, the Re and *R*_CT_ values for mwGO/RVC, CNT/RVC, and 9-CNT/mwGO/RVC composite electrodes are 17.4 Ω and 5686 Ω, 12.3 Ω and 2675 Ω, and 7.6 Ω and 1624 Ω, respectively. This indicates that the resistance value is reduced when CNT is coated on RVC instead of mwGO, and the lowest resistance value is observed when the mixture of both mwGO and CNT is used with RVC. It is attributed to the fact that the charge transfer is facilitated because of increased conductive paths due to CNT-CNT contacts/entanglements and CNT-mwGO network formation. The double-layer capacitance (*C*_DL_) value for the electrodes was calculated using the relation *w*_max_ = 1/(*R*_CT_*C*_DL_), where *w* represents the angular frequency. It was found 3.2 nF, 4.9 nF, and 6.3 nF for mwGO/RVC, CNT/RVC, and 9-CNT/mwGO/RVC, respectively. This increase in *C*_DL_ value after coating RVC with either CNT or 9-CNT/mwGO is due to the increase in surface area, which facilitates ion diffusion through the macro-porous structure of electrodes. The real part of impedance over low frequencies shows straight spike inclined around 45°, indicating the dominance of diffusion-limited Warburg impedance (Zw)/pseudo-capacitance (CP) because of limited mass transport of redox species from the bulk of solution to the electrode and ionic/charge accumulation on the surface of the electrode. It is observed in the figure that the inclination of straight spike is more for 9-CNT/mwGO/RVC electrode compared to others. This implies that 9-CNT/mwGO/RVC electrode has a higher value of pseudo-capacitance. This may be due to the presence of the carboxyl and carbonyl group on the surface of electrode materials.

In Fig. 5b, the real part of impedance (*Z*′) has been plotted against the angular frequency (*ω*) for mwGO/RVC, CNT/RVC, and 9-CNT/mwGO/RVC electrodes. This type of plotting is also known as Bode plot. It is observed that the impedance value of mwGO/RVC electrode is high over the whole frequency region compared to both CNT/RVC and 9-CNT/mwGO/RVC electrodes. This low value of impedance for CNT/RVC and 9-CNT/mwGO/RVC electrodes is due to the formation of preferential interconnected conductive networks on their surfaces that facilitate both the ionic and electronic transports. It has been shown diagrammatically in the inset of Fig. 5b how different parameters are correlated with the different regions of curve. Zw, *R*_CT_, *C*_DL_, and Re are identified over the low-frequency region inclined approximately 45°, over the middle frequency region inclined nearly 0° means horizontally, over the high-frequency region inclined nearly 90°, and finally over the extreme high-frequency region horizontally at 0°, respectively. Hence, it can be inferred that the result and diagram of both Nyquist and Bode plots are in good agreement with each other.

### Galvanostatic Charge-Discharge Process

**Fig. 6:**
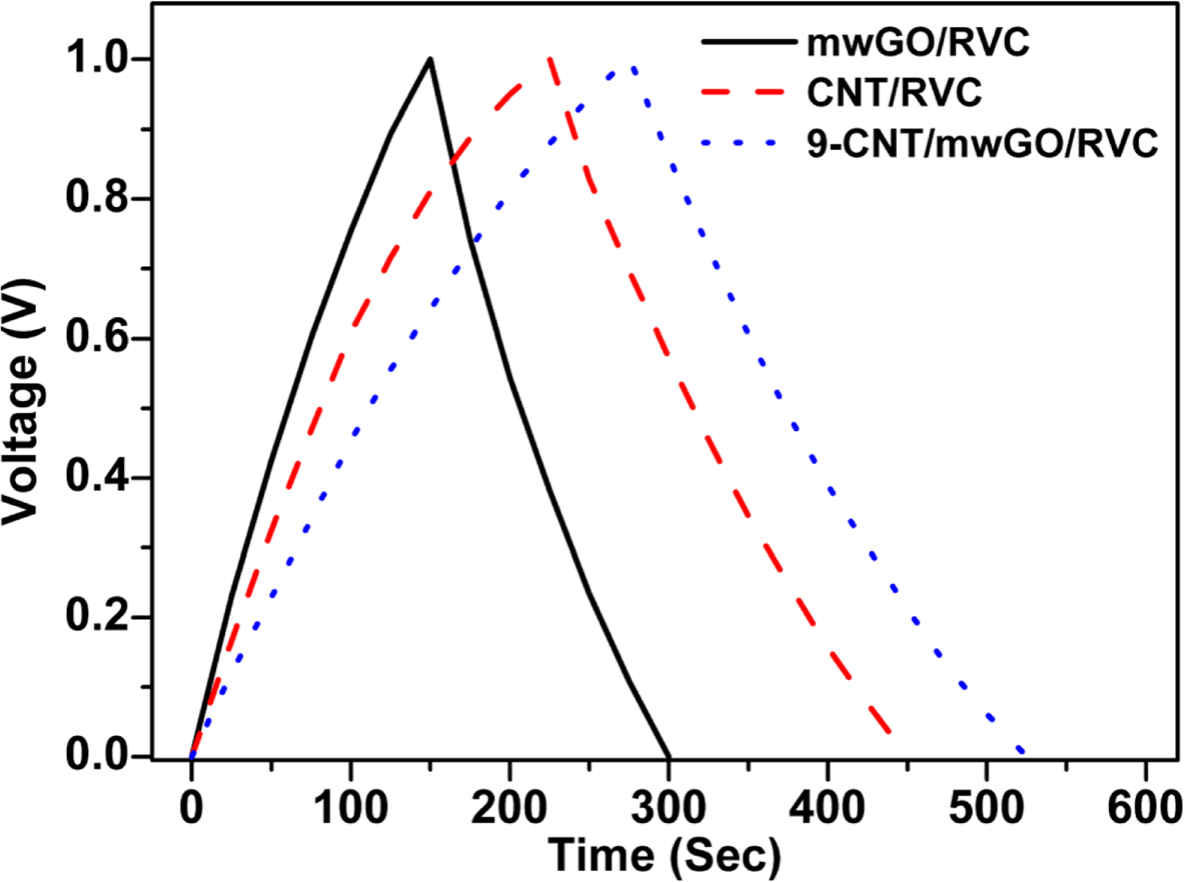
Galvanostatic charge-discharge (GCD) process of mwGO/RVC, CNT/RVC, and 9-CNT/mwGO/RVC electrodes

The galvanostatic charge-discharge (GCD) process of some selected composite electrodes measured within the voltage range 0–1 V and current density 0.2 A/g is presented in Fig. 6. It is observed in the figure that the charge and discharge (CD) curves of the electrodes are triangular in nature. The variation of voltage with respect to the time for all electrodes is almost linear. The behaviour of this type of curves is typical for carbon-based supercapacitor. The results also showed that all three electrodes have performed excellent super capacitive behaviour. It is seen from the figure that the CD time of CNT/RVC and 9-CNT/mwGO/RVC electrodes is higher compared to mwGO/RVC electrode. The CD time for 9-CNT/mwGO/RVC electrode is the highest amongst all electrodes. This can be attributed to the fact that more number of charge carriers are taking part in the CD process for CNT/RVC and 9-CNT/mwGO/RVC electrodes compared to mwGO/RVC electrode one. The symmetrical linear nature of the CD curves has demonstrated the formation of ideal electrical double-layer with the absence of any Faradic reaction. This implies that these carbon-based porous electrodes have good rate performance and fast ion transport mechanism. The electrode-specific capacitance can also be calculated from the discharge curve shown in Fig. 6 using the equation *C*sp = 2*Jt*/*V*, where Csp, *J*, *t*, and *V* represent specific capacitance, current density, discharge time, and voltage, respectively [1]. The calculated value of specific capacitance for mwGO/RVC, CNT/RVC and 9-CNT/mwGO/RVC are 85.74 F/g, 138.28 F/g, and 177.85 F/g, respectively. These results of specific capacitance are in almost good agreement with the results obtained through CV.

### Capacitance with Respect to Number of Cycle

**Fig. 7:**
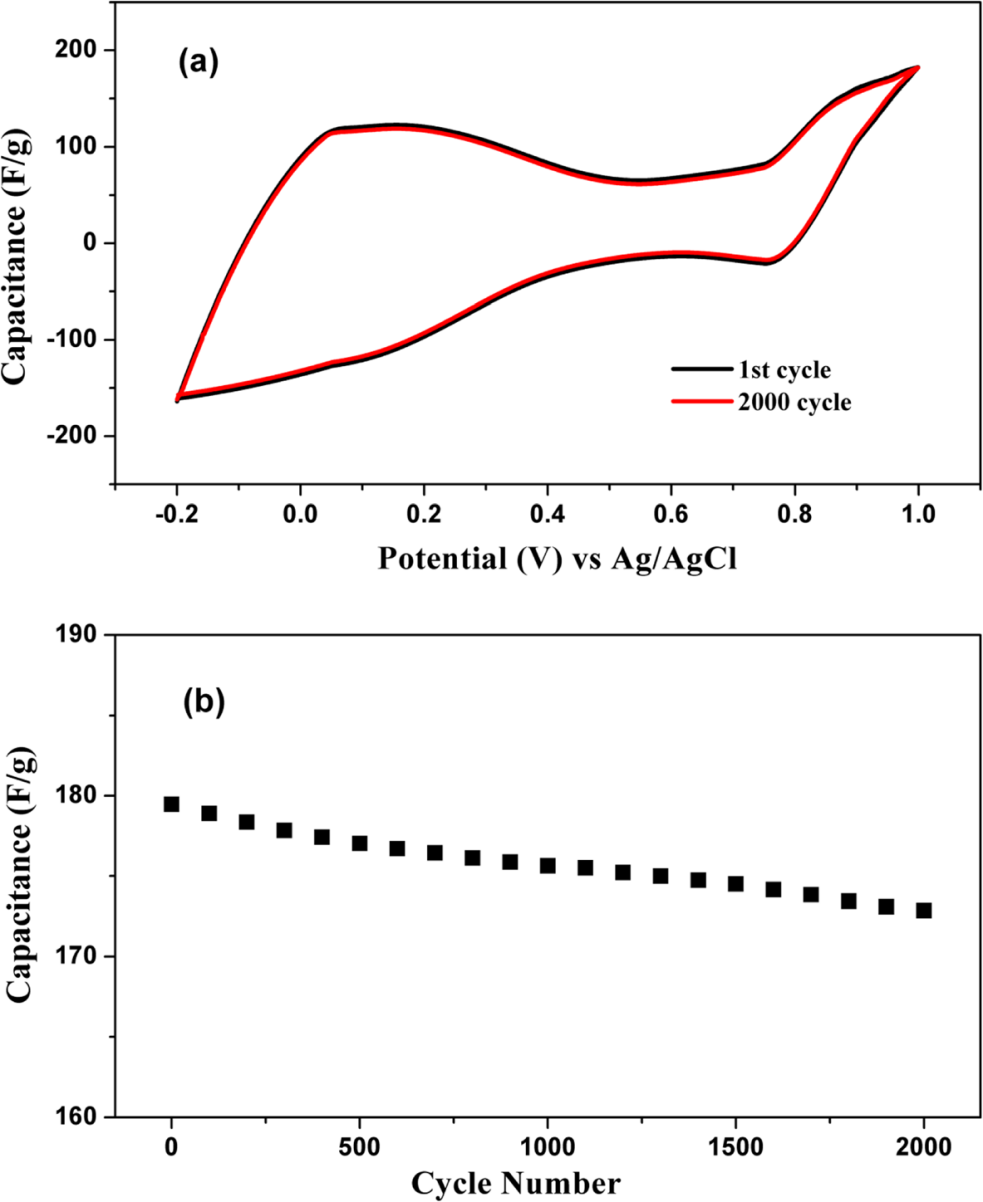
Capacitance against **a** potential and **b** number of cycle using 9-CNT/mwGO/RVC electrode measured at a scan rate of 5 mV/s

The variation of capacitance value with respect to the number of cycles for 9-CNT/mwGO/RVC composite electrode is presented in Fig. 7. In Fig. 7a, the redox curves of capacitance for only 1 and 2000 cycles are represented within the potential range − 0.2 to 1 V. Distorted rectangular shape of the curves is observed with 2 redox peaks because of the presence of oxygen-containing functional group in the composite electrode. The curves also show good cyclic performance. To check it precisely, as long cycle-life is required for high performance of electrode materials, the capacitance has been plotted against the number of cycles and shown in Fig. 7b. It is observed that the capacitance is decreasing with the increase in number of cycles. This decrement in capacitance value can be attributed to the decomposition of electrolyte after several cyclic tests. There is approximate 4% decrement in capacitance value after 2000 cycle of tests. Hence, it can be inferred that the electrode shows good cyclic stability.

## Conclusions

Graphene oxide nanosheets were successfully synthesized through a modified Hummer’s method and were reduced and exfoliated using microwave irradiation. The SEM images show significantly altered structure that is highly porous, forming an interconnected network with minimal restacking of the graphene. The XRD confirms the exfoliation, by microwave irradiation, of the GO due to the significant suppression of the sharp peak at 2*θ* = 10.8° and the increase in distance between graphene nanosheet layers in the individual stacks, while XPS spectra confirm that reduction of the GO is apparent due to the much sharper sp^2^ (C=C) at 284.4 eV peak. The D/G band ratio of the Raman spectra decreased from 1.26 to 1.08 signifying that the GO has undergone removal of some of its functional groups. In the novel CNT/mwGO/RVC electrodes, the CNT materials were found to be sandwiched amongst the graphene sheets that built a 3D highly porous architecture to facilitate rapid ion diffusion. These lead to enhanced van der Waals forces and friction. Moreover, the best electrochemical response with respect to capacitance and kinetic behaviour amongst all target electrodes concluded that the composite 9 a-SWCNT: 1 mwGO-coated RVC electrode (9-CNT/mwGO/RVC) performed as the best electrode. It showed specific capacitance value 179.39 F/g that means 29% increment compared to the CNT/RVC electrode. This optimal electrode also had a very high cyclic voltammogram (CV) curve stability that maintained an electrochemical cycling stability of 97% after 2000 cycles. The results indicate that the electrodes can be efficiently used as the electrode materials in capacitive deionization cell for purifying water.

## Supplementary information

**Additional file 1: Figure S1.** Scheme for the preparation process of composite a-SWCNT/mwGO coating solution. **Figure S2.** (a,b,c) Photo images and (d,e,f) SEM micrographs of 60, 45 and 30 ppi RVC samples, respectively. (g) The specific capacitance of a-SWCNT coated RVC electrodes of various porosities in 1 M NaCl solution calculated from cyclic voltammograms recorded in a voltage range between -0.2 to 1.0 V using a three-electrode system vs Ag/AgCl at 5mV/s scan rate. **Figure S3.** Schematic diagram of the preparation process of ratio 9:1 composite a-SWCNT/mwGO coated RVC electrode. **Figure S4.** (a and b) Energy-dispersive X-ray (EDX) spectra, (c) the XRD patterns of the graphite flakes powder and GO film respectively, and (d) Fourier-Transform Infrared (FT-IR) spectrum for graphene oxide film. Inset shows SEM and optical images of the (a) graphite flakes powder and (b) the GO film. **Figure S5.** (a and b) SEM images of GO and mwGO, respectively, (c) the XRD patterns of GO and mwGO and (d and e) Energy-dispersive X-ray (EDX) spectrum of GO film and microwave irradiated graphene oxide. Inset shows optical images of the (d) GO film and (e) mwGO. **Figure S6.** Raman spectroscopy of flake graphite, GO and mwGO. **Figure S7.** XPS spectra: comparison of the C1s spectra for (a) GO and (b) microwave irradiated graphene oxide (mwGO). **Figure S8.** Thermogravimetric analysis (TGA) of graphite flakes, GO, and microwave irradiated graphene oxide. **Figure S9.** (a) Visible absorption spectra vs wavelength of 0.1% w/v mwGO dispersion, where inset photographs are for 0.1% w/v mwGO before sonication and after 35 minutes sonication, and (b) Absorbance at 660 nm *vs* sonication time. Arrow in (a) is indicating the direction of increasing sonication time. (c) Sonication energy vs absorbance at 660 nm for 0.1 % w/v mwGO dispersions.

## Data Availability

The datasets that supports our results and conclusions are included within this article and supplementary files.

## Abbreviations

2D: Two-dimensional
3D: Three-dimensional
AR: Analytical reagent
C_2_H_6_O: Ethanol
CDI: Capacitive deionization
CE: Counter electrode
CH_3_OH: Methanol
CNT: Carbon nanotubes
CV: Cyclic voltammetry
DMF: Dimethyl formamide
EDX: Energy-dispersive X-ray spectra
EIS: Electrostatic impedance spectra
FESEM: Field emission scanning electron microscopy
GCD: Galvanostatic charge-discharge
H_2_O_2_: Hydrogen peroxide
H_2_SO_4_: Sulphuric acid
HCl: Hydrochloric acid
HNO_3_: Nitric acid
KMNO_4_: Potassium permanganate
Mn(OH)_2_: Manganese hydroxide
mwGO: Microwave-irradiated graphene oxide
NaCl: Sodium chloride
PEDOT: Polyethylene dioxylthiophene
PPy: Polypyrrole
RVC: Reticulated vitreous carbon
SWCNTs: Single-walled carbon nanotubes
TGA: Thermogravimetric analysis
TX: Texus
UV-VIS: Ultra-violet visible
WE: Working electrode
XPS: X-ray photoelectron spectra
XRD: X-ray diffraction
