# Supplementary material for: Performance Enhancement of Modified 3D SWCNT/RVC Electrodes Using Microwave-Irradiated Graphene Oxide
Source: Nanoscale Res Lett. 2019 Nov 27;14:351. doi: 10.1186/s11671-019-3174-9 (PMC6881498; doi:10.1186/s11671-019-3174-9)
Supplement: Supplementary file 1 — Additional file 1: Figure S1. Scheme for the preparation process of composite a-SWCNT/mwGO coating solution. Figure S2. (a,b,c) Photo images and (d,e,f) SEM micrographs of 60, 45 and 30 ppi RVC samples, respectively. (g) The specific capacitance of a-SWCNT coated RVC electrodes of various porosities in 1 M NaCl solution calculated from cyclic voltammograms recorded in a voltage range between -0.2 to 1.0 V using a three-electrode system vs Ag/AgCl at 5mV/s scan rate. Figure S3. Schematic diagram of the preparation process of ratio 9:1 composite a-SWCNT/mwGO coated RVC electrode. Figure S4. (a and b) Energy-dispersive X-ray (EDX) spectra, (c) the XRD patterns of the graphite flakes powder and GO film respectively, and (d) Fourier-Transform Infrared (FT-IR) spectrum for graphene oxide film. Inset shows SEM and optical images of the (a) graphite flakes powder and (b) the GO film. Figure S5. (a and b) SEM images of GO and mwGO, respectively, (c) the XRD patterns of GO and mwGO and (d and e) Energy-dispersive X-ray (EDX) spectrum of GO film and microwave irradiated graphene oxide. Inset shows optical images of the (d) GO film and (e) mwGO. Figure S6. Raman spectroscopy of flake graphite, GO and mwGO. Figure S7. XPS spectra: comparison of the C1s spectra for (a) GO and (b) microwave irradiated graphene oxide (mwGO). Figure S8. Thermogravimetric analysis (TGA) of graphite flakes, GO, and microwave irradiated graphene oxide. Figure S9. (a) Visible absorption spectra vs wavelength of 0.1% w/v mwGO dispersion, where inset photographs are for 0.1% w/v mwGO before sonication and after 35 minutes sonication, and (b) Absorbance at 660 nm vs sonication time. Arrow in (a) is indicating the direction of increasing sonication time. (c) Sonication energy vs absorbance at 660 nm for 0.1 % w/v mwGO dispersions. [file 11671_2019_3174_MOESM1_ESM.docx]

**Supplementary Information: Performance Enhancement of Modified 3D SWCNT/RVC Electrodes using Microwave Irradiated Graphene Oxide**

**Ali Aldalbahi^1^*, Mostafizur Rahaman^1^*, Mohammed Almoiqli^2^**

^1^Department of Chemistry, College of Science, King Saud University, Riyadh 11451, Saudi Arabia

^2^Nuclear Sciences Research Institute, King Abdulaziz City for Science and Technology, Riyadh 11442, Saudi Arabia

***Corresponding Author:** aaldalbahi@ksu.edu.sa (Ali Aldalbahi); mrahaman@ksu.edu.sa (M. Rahaman)

**
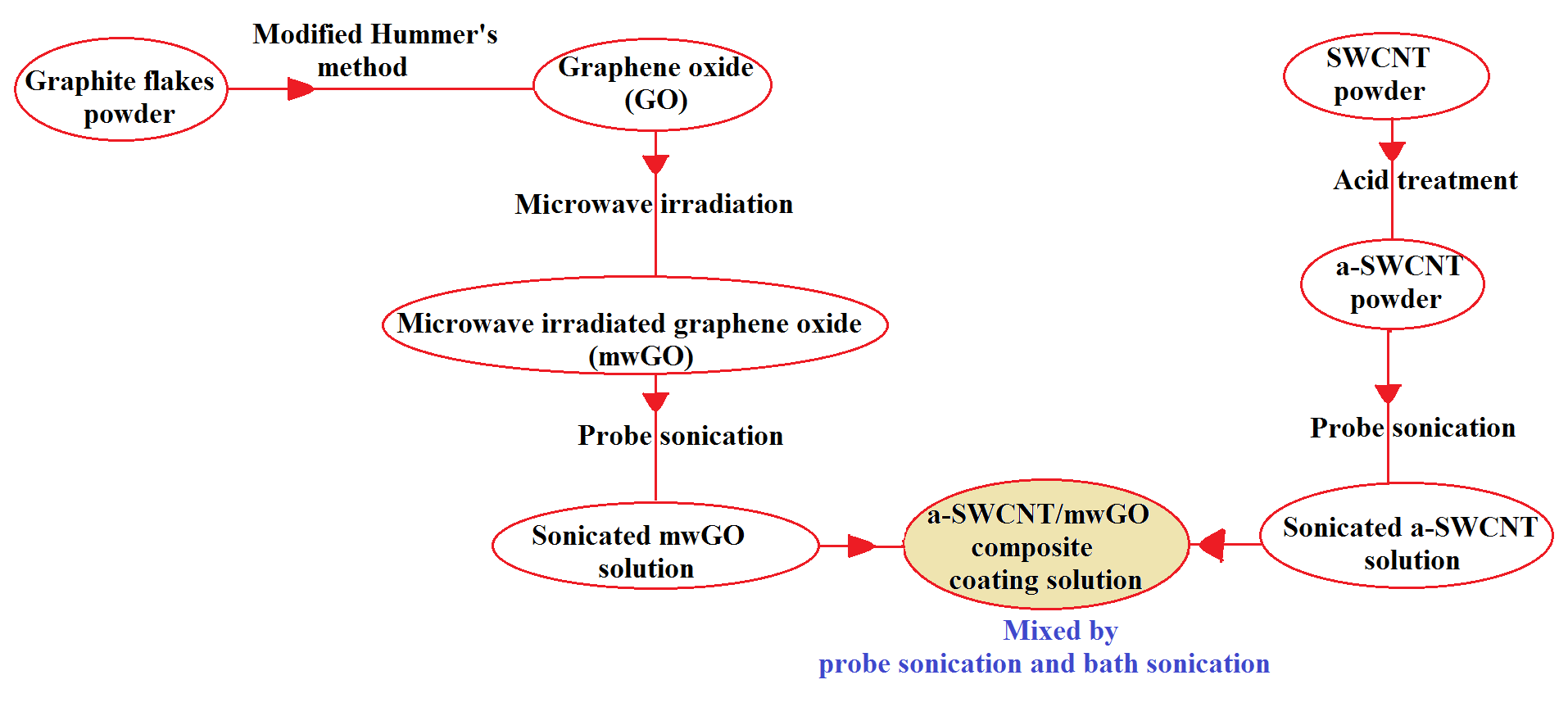
**

**Figure S1.** Scheme for the preparation process of composite a-SWCNT/mwGO coating solution.

**S2. Optimization of RVC electrodes coated with a-SWCNT**

The general purpose of this work is the optimization of the reticulated vitreous carbon (RVC) electrodes of different porosities coated with a-SWCNT. RVC electrode has a free void volume between 90% and 97%. Thus, RVC electrodes have a low flow resistance. Figure S2 (a, b, and c) shows photo images of three RVC electrodes with porosities of 60, 45 and 30 ppi (nominal pores per inch). It is clear that the free void volume of the RVC electrodes increases with decreasing ppi grade. The average pore sizes of the RVC electrodes were calculated from SEM images by measuring the distance between green lines in Figure S2 (d, e, and f) and they were 350, 700 and 900 µm for 60, 45 and 30 ppi, respectively. RVC electrode properties are dependent on the ppi grade. If the amount of pores per inch (ppi) increases, the electrode area per unit electrode volume will increase as well. According to previous reported properties of RVC [1, 2], which are good surface area, conductivity and good mechanical strength, it is envisaged that the RVC electrode that has the largest number of pores per inch would be the best electrode for use in a CDI system. Therefore, in order to confirm that the smaller pores electrode is the best electrode for loading of a-SWCNT for use in a CDI system, we investigated the influence of all electrode capacitances of RVC electrodes with different pore sizes coated with the same amount of a-SWCNT. All RVC electrodes had the same geometric volume (dimensions of 4.0cm*1.8cm*0.3cm) and the same amount of a-SWCNT was coated, around 6 mg. The effect of different pores per inch was investigated in aqueous solution. Figure S2 (g) shows the capacitances of all at the potential scan rate of 5 mV/s. It can be seen that the highest specific capacitance was 267.24 F/ g for a-SWCNT coated RVC electrode with 60 pores per inch, and the specific capacitance decreased with a decrease in the amount of pores per inch. This is because the RVC electrode with 60 ppi has higher surface area per volume of electrode. In conclusion, therefore, 60 ppi RVC electrodes were selected as substrates to load a-SWCNT for use as electrodes.


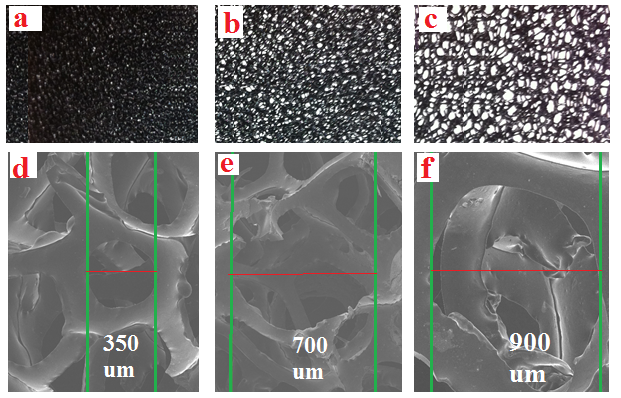

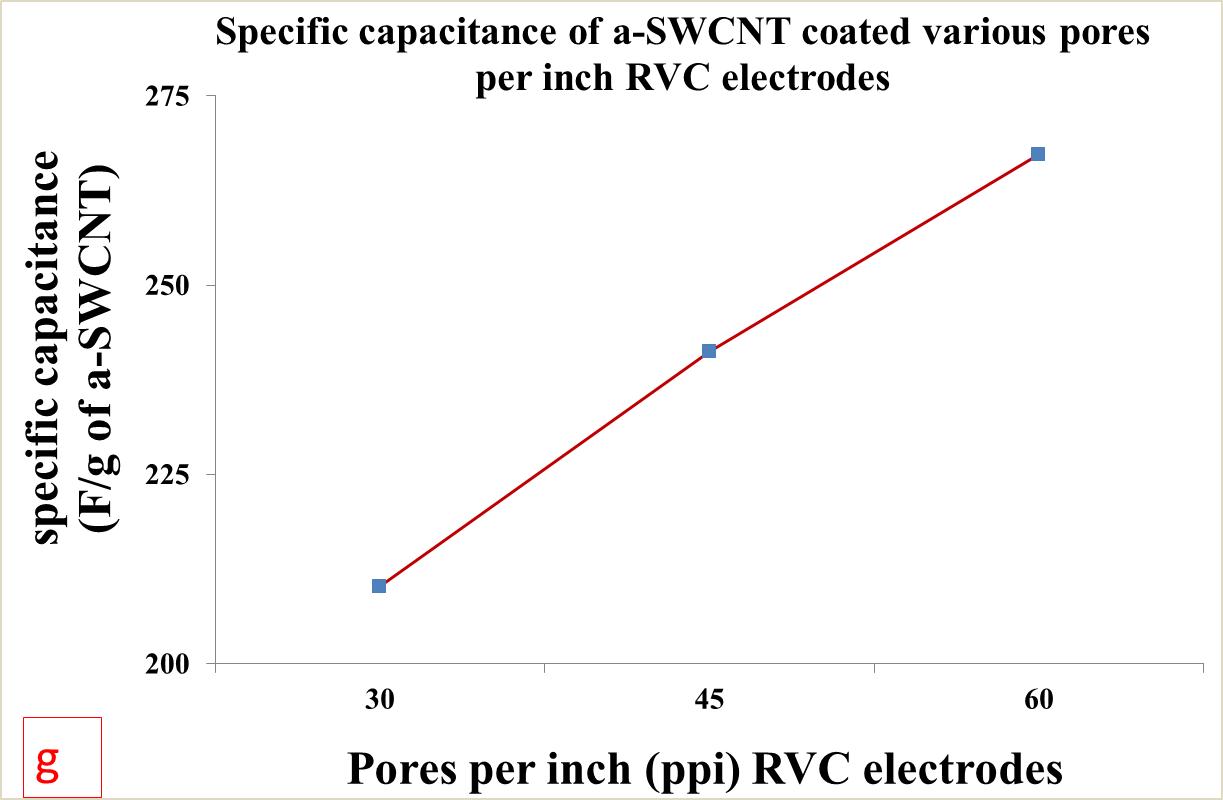


**Figure S2.** (a,b,c) Photo images and (d,e,f) SEM micrographs of 60, 45 and 30 ppi RVC samples, respectively. (g) The specific capacitance of a-SWCNT coated RVC electrodes of various porosities in 1 M NaCl solution calculated from cyclic voltammograms recorded in a voltage range between -0.2 to 1.0 V using a three-electrode system vs Ag/AgCl at 5mV/s scan rate.


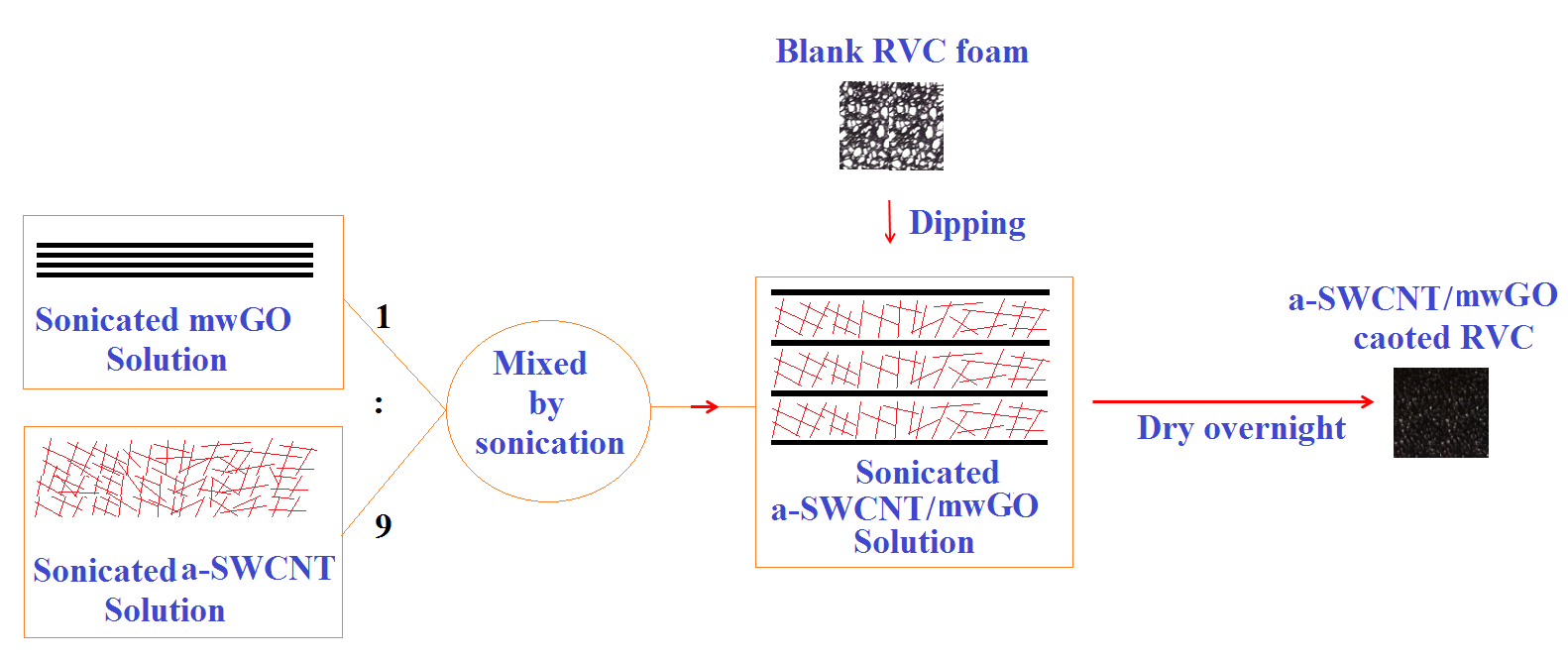


**Figure S3.** Schematic diagram of the preparation process of ratio 9:1 composite a-SWCNT/mw GO coated RVC electrode.


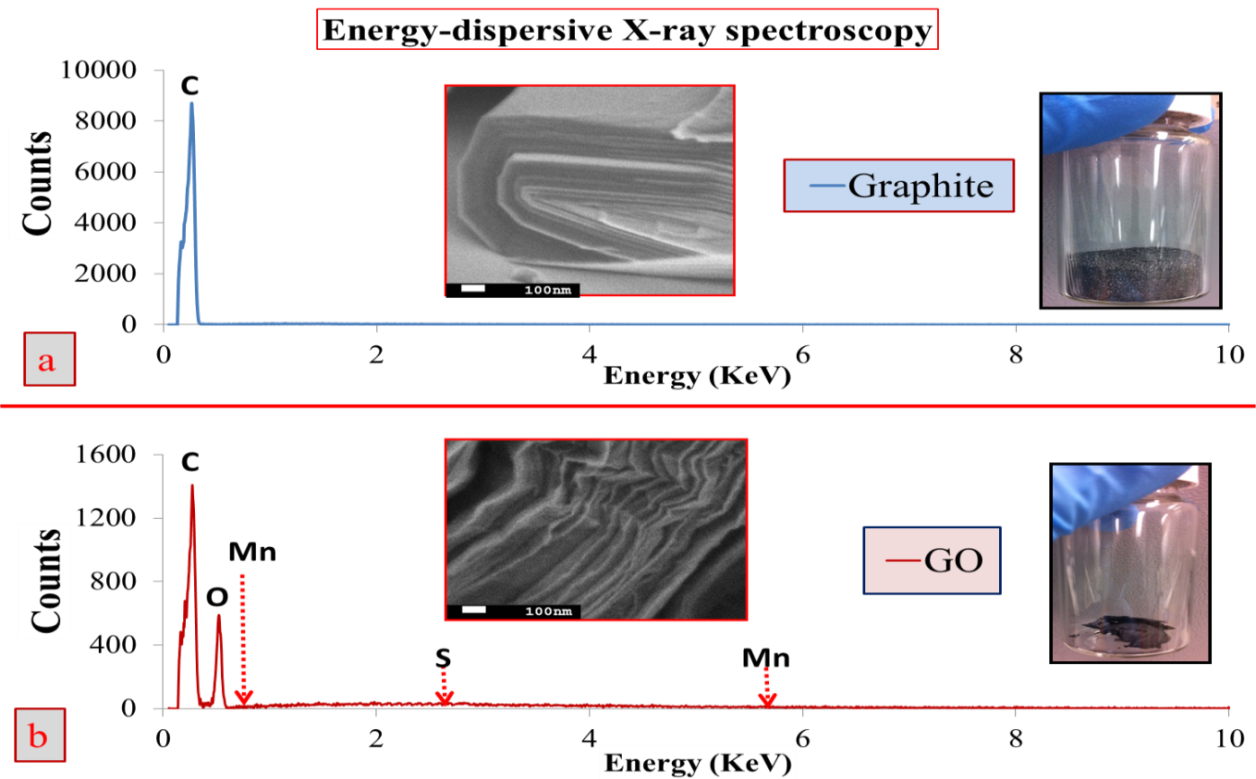

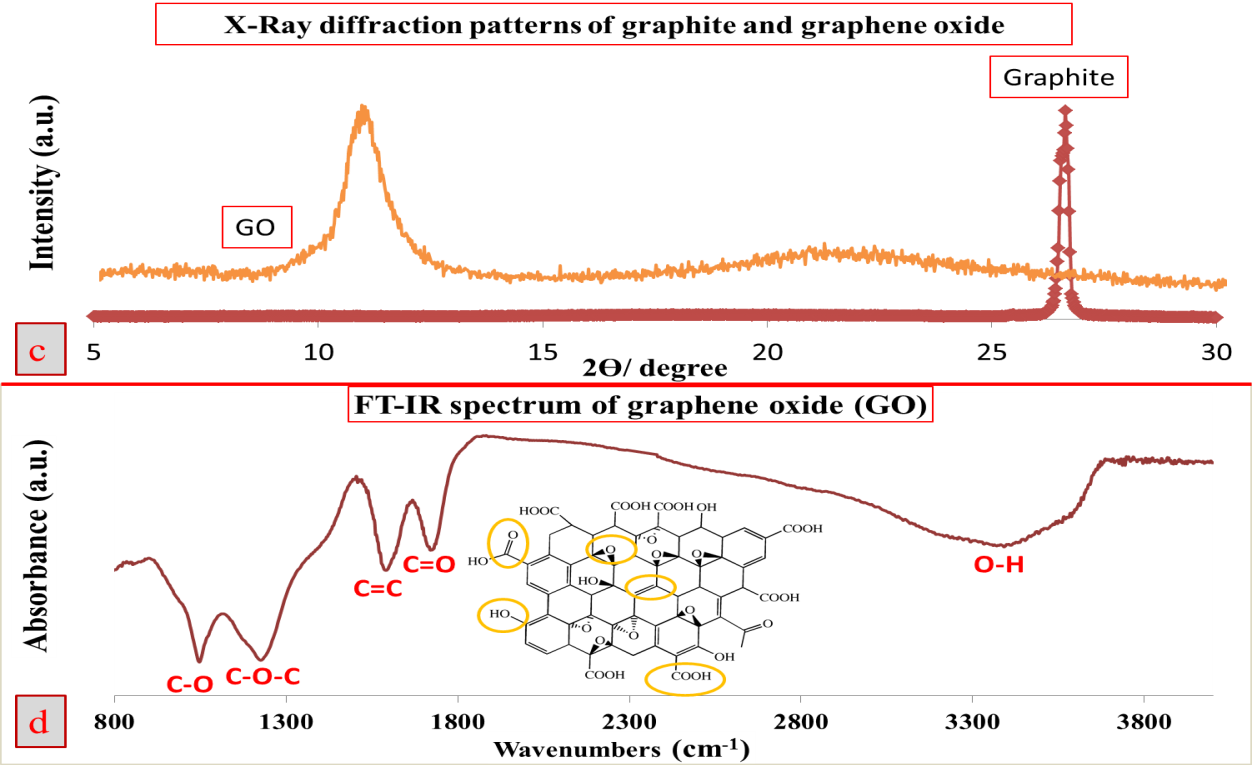


**Figure S4.** (a and b) Energy-dispersive X-ray (EDX) spectra, (c) the XRD patterns of the graphite flakes powder and GO film respectively, and (d) Fourier-Transform Infrared (FT-IR) spectrum for graphene oxide film. Inset shows SEM and optical images of the (a) graphite flakes powder and (b) the GO film.


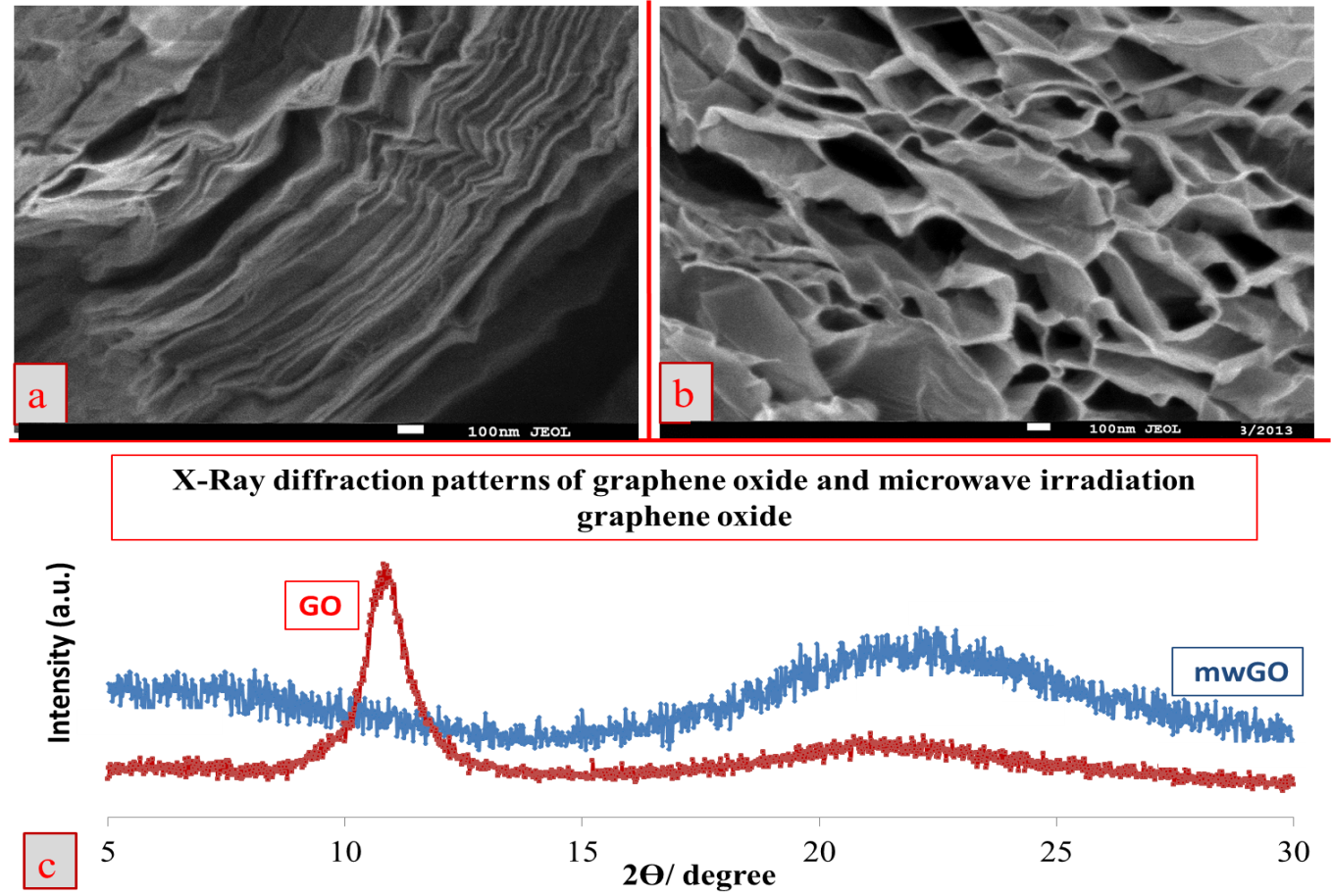

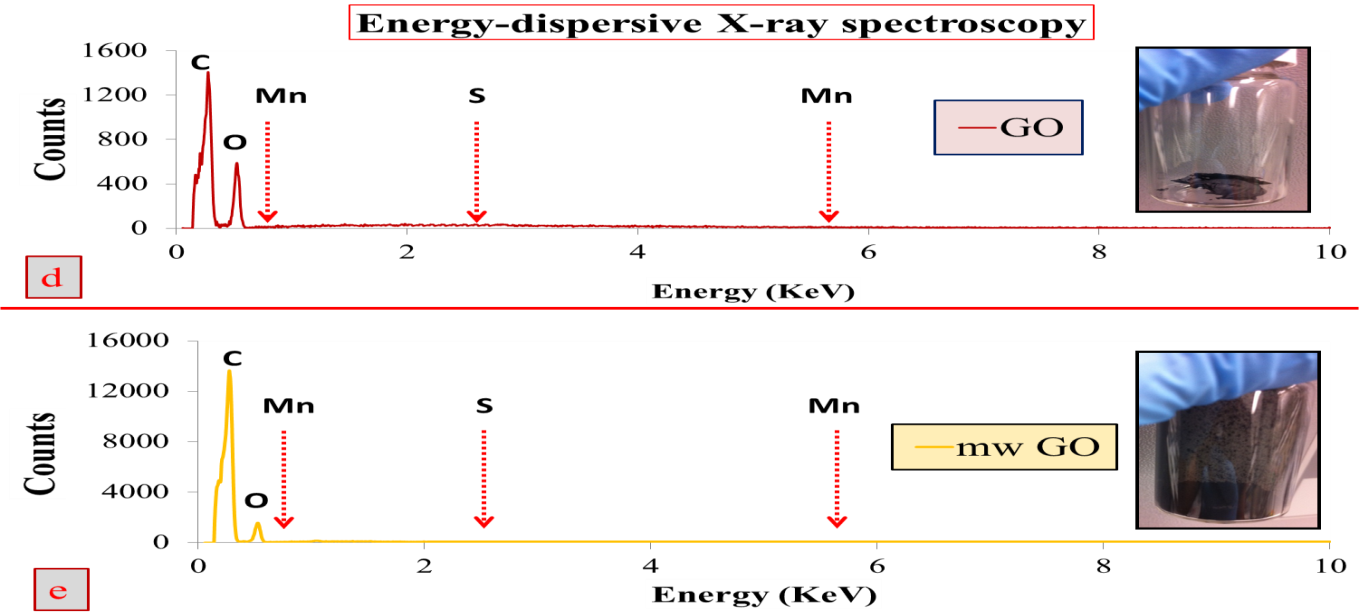


**Figure S5.** (a and b) SEM images of GO and mwGO, respectively, (c) the XRD patterns of GO and mwGO and (d and e) Energy-dispersive X-ray (EDX) spectrum of GO film and microwave irradiated graphene oxide. Inset shows optical images of the (d) GO film and (e) mwGO.


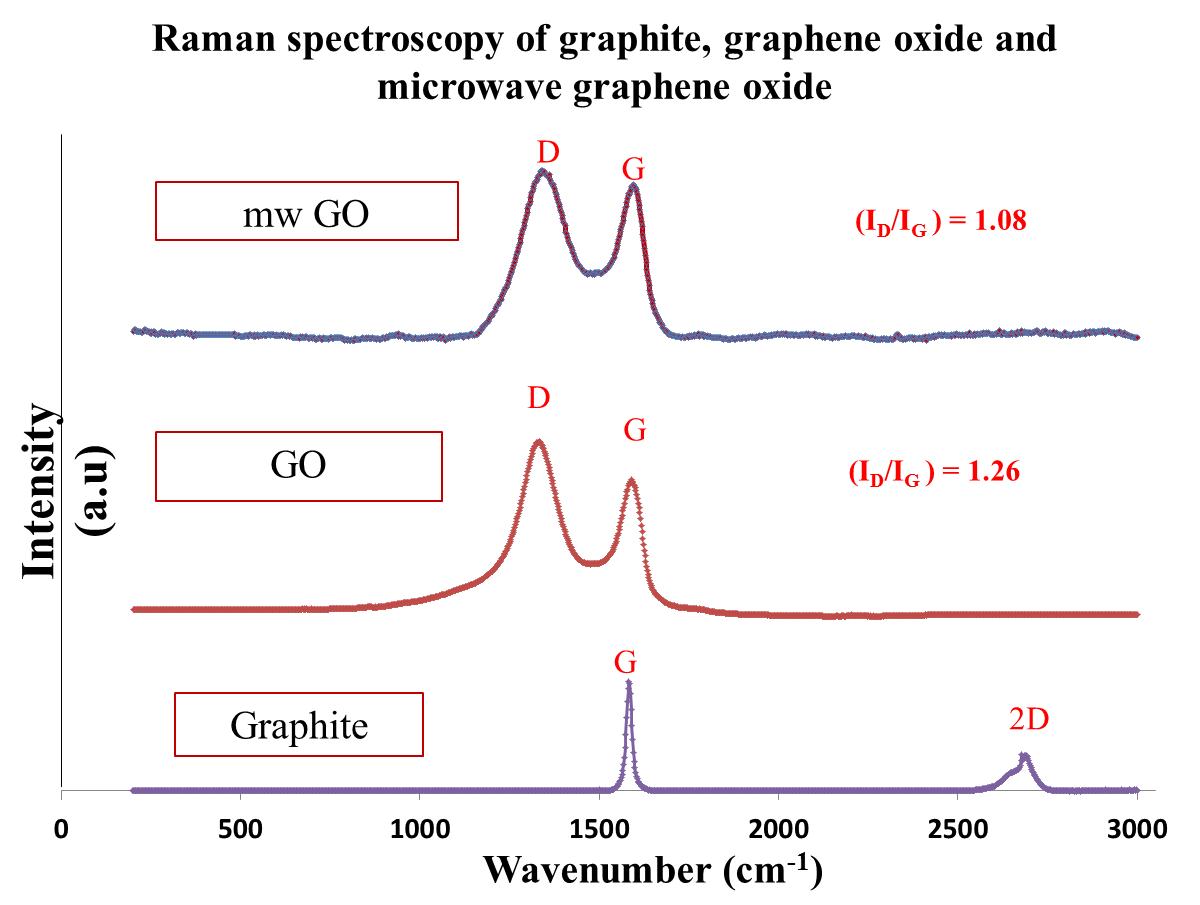


**Figure S6.** Raman spectroscopy of flake graphite, GO and mwGO.


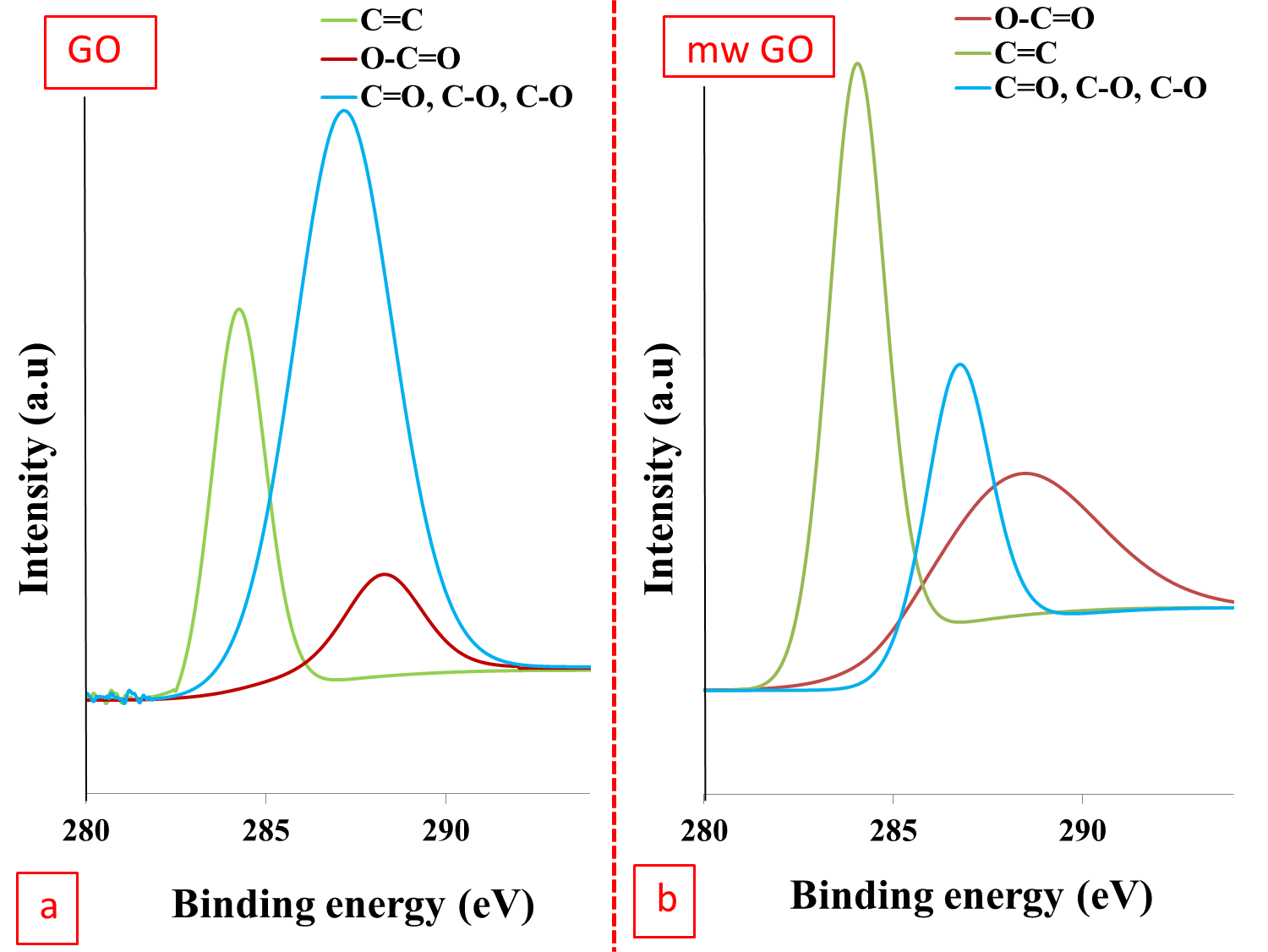


**Figure S7.** XPS spectra: comparison of the C1s spectra for (a) GO and (b) microwave irradiated graphene oxide (mwGO).


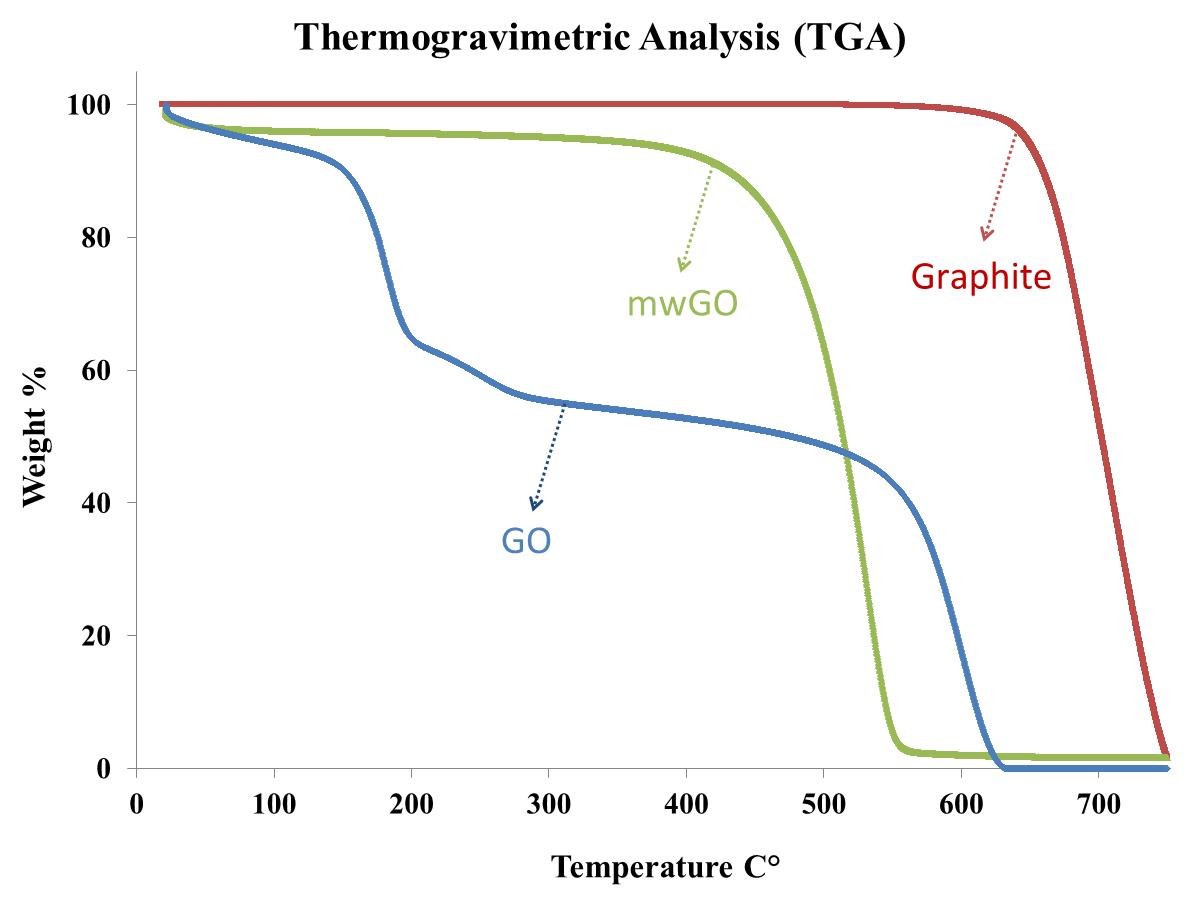


**Figure S8.** Thermogravimetric analysis (TGA) of graphite flakes, GO, and microwave irradiated graphene oxide.

**Figure S9.** (a) Visible absorption spectra vs wavelength of 0.1% w/v mwGO dispersion, where inset photographs are for 0.1% w/v mwGO before sonication and after 35 minutes sonication, and (b) Absorbance at 660 nm *vs* sonication time. Arrow in (a) is indicating the direction of increasing sonication time. (c) Sonication energy vs absorbance at 660 nm for 0.1 % w/v mwGO dispersions.


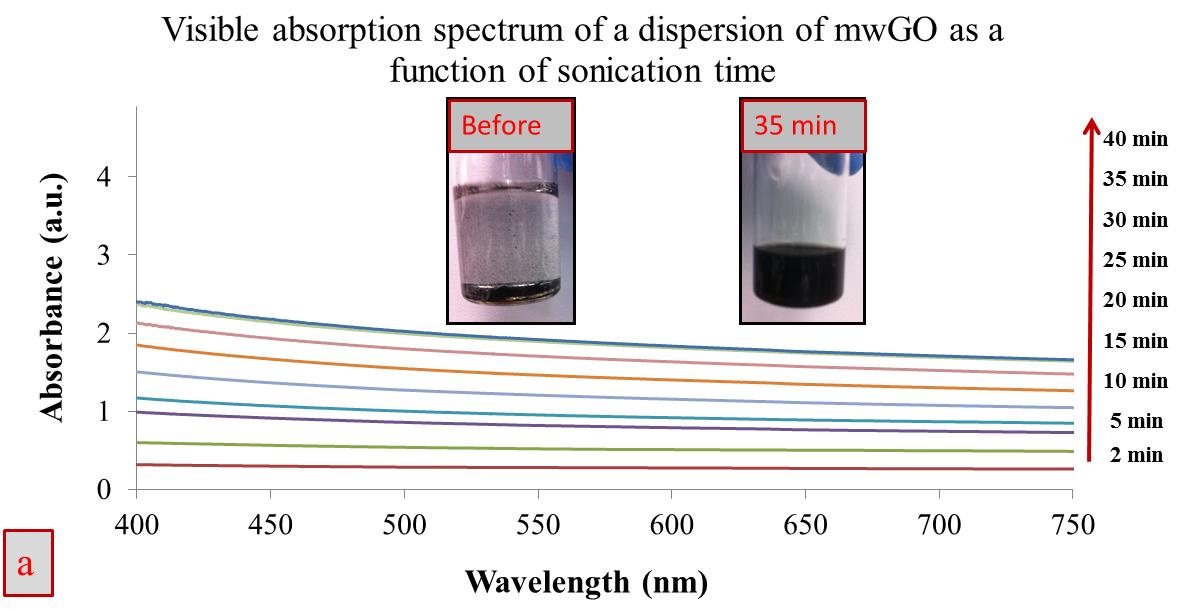

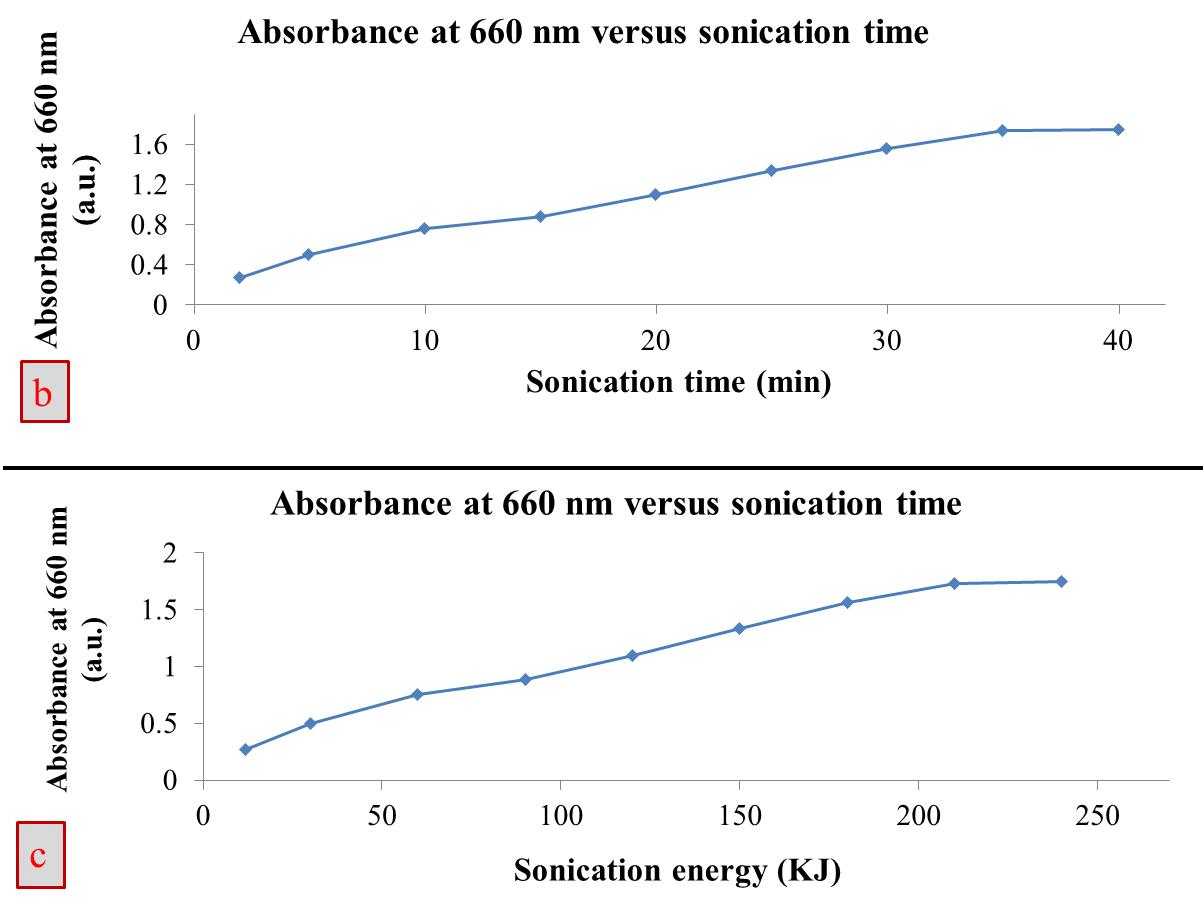


**References**

[1] Friedrich, J.M., *Reticulated vitreous carbon as an electrode material.* Journal of Electroanalytical Chemistry and Interfacial Electrochemistry, 2004. **561**(1): p. 203-217.

[2] Tondi, G., V. Fierro, A. Pizzi and A. Celzard, *Tannin-based carbon foams.* Carbon, 2009. **47**(6): p. 1480-1492.
